# Identification of isoAsp7-Aβ as a major Aβ variant in Alzheimer’s disease, dementia with Lewy bodies and vascular dementia

**DOI:** 10.1007/s00401-024-02824-9

**Published:** 2024-12-03

**Authors:** Sarah Schrempel, Anna Katharina Kottwitz, Anke Piechotta, Kathrin Gnoth, Luca Büschgens, Maike Hartlage-Rübsamen, Markus Morawski, Mathias Schenk, Martin Kleinschmidt, Geidy E. Serrano, Thomas G. Beach, Agueda Rostagno, Jorge Ghiso, Michael T. Heneka, Jochen Walter, Oliver Wirths, Stephan Schilling, Steffen Roßner

**Affiliations:** 1 Paul Flechsig Institute – Centre of Neuropathology and Brain Research, University of Leipzig, Liebigstraße 19, 04103 Leipzig, Germany; 2 Department of Molecular Drug Design and Target Validation, Fraunhofer Institute for Cell Therapy and Immunology, 06120 Halle (Saale), Germany; 3 Center for Natural Product-based Therapeutics, Anhalt University of Applied Sciences, 06366 Köthen, Germany; 4 Department of Psychiatry and Psychotherapy, University Medical Center Göttingen, Georg-August-University, 37075 Göttingen, Germany; 5 Civin Laboratory for Neuropathology, Brain and Body Donation Program, Banner Sun Health Research Institute, 10515 W Santa Fe Drive, Sun City, AZ 85351 USA; 6 Department of Pathology, New York University School of Medicine, New York, NY USA; 7 Luxembourg Centre for Systems Biomedicine, University of Luxembourg, Belval, Luxembourg; 8 Center of Neurology, Molecular Cell Biology, University Hospital Bonn, 53127 Bonn, Germany

**Keywords:** Amyloid-β, Abeta, Post-translational modifications, Alzheimer’s disease, Vascular dementia, Dementia with Lewy bodies, Mini Mental State Examination, Automated histology quantification

## Abstract

The formation of amyloid-β (Aβ) aggregates in brain is a neuropathological hallmark of Alzheimer’s disease (AD). However, there is mounting evidence that Aβ also plays a pathogenic role in other types of dementia and that specific post-translational Aβ modifications contribute to its pathogenic profile. The objective of this study was to test the hypothesis that distinct types of dementia are characterized by specific patterns of post-translationally modified Aβ variants. We conducted a comparative analysis and quantified Aβ as well as Aβ with pyroglutamate (pGlu3-Aβ and pGlu11-Aβ), N-truncation (Aβ(4-X)), isoaspartate racemization (isoAsp7-Aβ and isoAsp27-Aβ), phosphorylation (pSer8-Aβ and pSer26-Aβ) or nitration (3NTyr10-Aβ) modification in *post mortem* human brain tissue from non-demented control subjects in comparison to tissue classified as pre-symptomatic AD (Pre-AD), AD, dementia with Lewy bodies and vascular dementia. Aβ modification-specific immunohistochemical labelings of brain sections from the posterior superior temporal gyrus were examined by machine learning-based segmentation protocols and immunoassay analyses in brain tissue after sequential Aβ extraction were carried out. Our findings revealed that AD cases displayed the highest concentrations of all Aβ variants followed by dementia with Lewy bodies, Pre-AD, vascular dementia and non-demented controls. With both analytical methods, we identified the isoAsp7-Aβ variant as a highly abundant Aβ form in all clinical conditions, followed by Aβ(4-X), pGlu3-Aβ, pGlu11-Aβ and pSer8-Aβ. These Aβ variants were detected in distinct plaque types of compact, coarse-grained, cored and diffuse morphologies and, with varying frequencies, in cerebral blood vessels. The 3NTyr10-Aβ, pSer26-Aβ and isoAsp27-Aβ variants were not found to be present in Aβ plaques but were detected intraneuronally. There was a strong positive correlation between isoAsp7-Aβ and Thal phase and a moderate negative correlation between isoAsp7-Aβ and performance on the Mini Mental State Examination. Furthermore, the abundance of all Aβ variants was highest in APOE 3/4 carriers. In aggregation assays, the isoAsp7-Aβ, pGlu3-Aβ and pGlu11-Aβ variants showed instant fibril formation without lag phase, whereas Aβ(4-X), pSer26-Aβ and isoAsp27-Aβ did not form fibrils. We conclude that targeting Aβ post-translational modifications, and in particular the highly abundant isoAsp7-Aβ variant, might be considered for diagnostic and therapeutic approaches in different types of dementia. Hence, our findings might have implications for current antibody-based therapies of AD.

**Supplementary Information:**

The online version contains supplementary material available at 10.1007/s00401-024-02824-9.

## Introduction

Alzheimer’s disease (AD) and other types of dementia are characterized by the degeneration of defined subsets of neurons and by the deposition of proteins that accumulate as amyloid plaques, neurofibrillary tangles, Lewy bodies and glial cytoplasmic inclusions. Historically, the aggregated proteins were assigned to defined clinical conditions: Aβ and Tau to AD [19, 37], α-synuclein to the synucleinopathies Parkinson’s disease (PD) [86, 110], dementia with Lewy bodies (DLB) [9, 110] and multiple system atrophy (MSA) [5, 116] and Huntingtin to Huntington’s disease (HD) [64, 102]. This constricted perspective has changed in the last two decades when proteins formerly considered typical for a specific clinical condition were also observed in other types of pathology [4, 6, 14, 34, 42, 53, 117, 118]. Therefore, we became interested in analyzing the pathology of distinct Aβ peptide variants in brains from different types of dementia: AD, DLB and vascular dementia (VAD) and, at an early disease stage, Pre-AD.

It is well established that Aβ per se is a physiological peptide with cellular functions. For example, it was shown to be critical for neuronal survival [82]. Moreover, Aβ acts as a positive modulator of neurotransmitter release probability in hippocampal synapses [1] and its hippocampal production is enhanced during memory induction in experimental mouse models [84]. In addition, Aβ concentrations measured by ELISA in brain interstitial fluid have been shown to correlate with neurological status in human subjects [20]. On one hand, in the clinical condition of familial AD, amyloid precursor protein overexpression and mutations or mutations of its processing γ-secretase, respectively, lead to pathological Aβ accumulation [87, 121, 126]. In sporadic AD, on the other hand, the exact mechanisms leading to Aβ accumulation are not known, but may include a pathological formation of Aβ post-translational modifications (PTMs), such as N-terminal truncation [17, 66], pyroglutamylation [63, 97], phosphorylation [55, 57], nitration [61] and isoaspartate formation [107, 115] (for review, see [62, 91]). Such Aβ PTMs might be valuable diagnostic markers and also therapeutic targets for pharmacologic interventions. In case of enzyme-catalyzed generation of PTMs, their formation could be prevented by enzyme inhibition and already formed Aβ PTMs could be targeted by specific monoclonal antibodies. A good example for this strategy is the prevention of pGlu3-Aβ formation by inhibition of glutaminyl cyclase with Varoglutamstat [100, 119] and clearance of existing pGlu3-Aβ by the antibody Donanemab [40, 108].

**Fig. 1:**
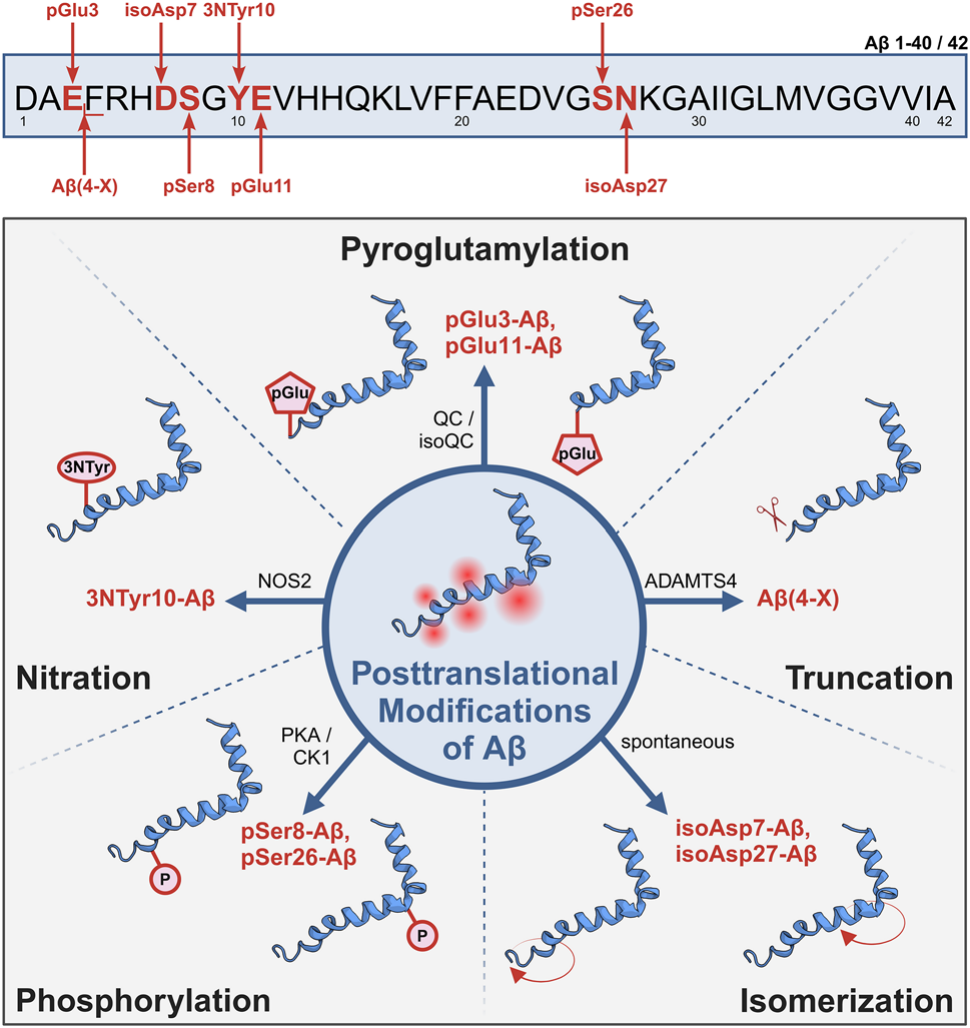
Schematic representation of the Aβ peptide variants investigated in the present study in brain tissue of deceased patients categorized in Pre-AD, AD, DLB, VAD and control subjects. Together, eight different Aβ variants from five groups of post-translational modifications were analyzed side-by-side in a comprehensive manner by immunohistochemical and biochemical methods. Aβ structure from [25]. *Created with BioRender.com*

However, to date, there is no comprehensive side-by-side analysis of all the above-mentioned Aβ variants in different types of dementia, which would allow the identification of a therapeutic target for a specific type of dementia or a combination of dementia diseases. Therefore, we here asked the question whether the different clinical conditions of dementia under investigation are characterized by specific signatures of Aβ PTMs or display similar patterns of these Aβ variants (Fig. 1). To address this issue, we used well-preserved human brain tissue with a short *post mortem* delay and a detailed clinical and pathological characterization that enabled us to correlate clinical with histopathological and biochemical findings.

## Materials and methods

**Fig. 2:**
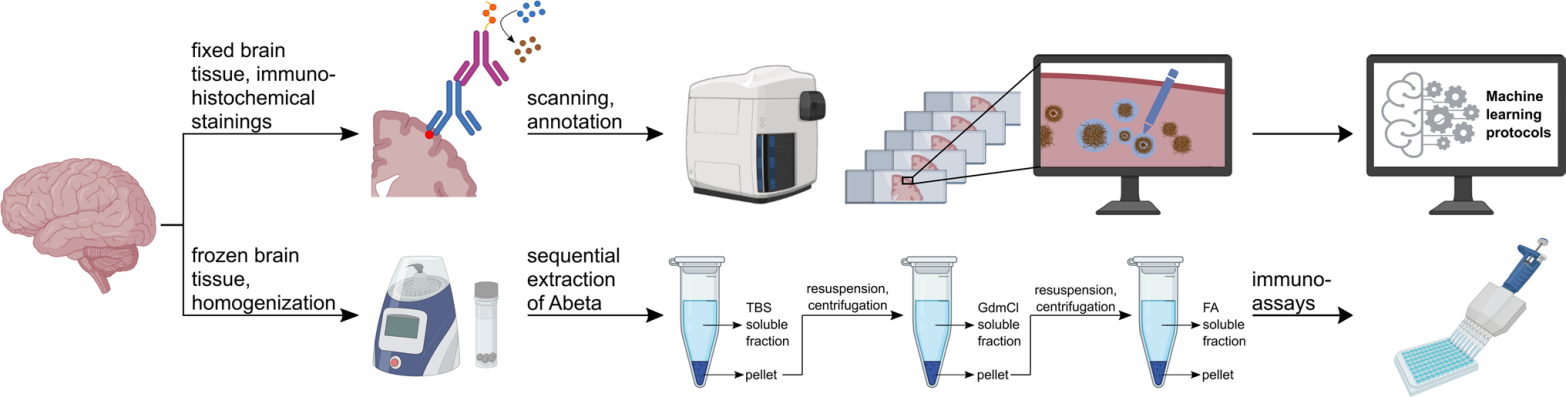
Schematic presentation of the workflow for the immunohistochemical (top) and biochemical (bottom) analyses of Aβ peptide variants in human brain tissue from Pre-AD, AD, DLB, VAD and control subjects. The immunohistochemically labeled brain slices were digitized with an Axioscan slide scanner and subjected to plaque load quantification by machine learning-based segmentation protocols. For biochemical analyses, Aβ peptides were extracted from unfixed human brain tissue by sequential centrifugation of Tris-buffered saline (TBS), guanidinium chloride (GdmCl) and formic acid (FA) dilutions followed by quantification by immunoassays using specific monoclonal antibodies. *Created with BioRender.com*

The general workflow of the immunohistochemical and biochemical analyses conducted in the present study is summarized in Fig. 2. Human brain tissue from all cases was available to be used for both analytical methods.

### Human brain tissue

The human brain tissue was provided by the Arizona Study of Aging and Neurodegenerative Disorders (AZSAND) and Brain and Body Donation Program of Banner Sun Health Research Institute in Sun City, Arizona. Case recruitment and autopsy were performed as approved by institutional review boards overseeing AZSAND’s Brain and Body Donation Program [13]. The required consent was obtained for all cases. The diagnosis and staging of AD, DLB and VAD for the cases used in this study were based on standardized clinicopathological criteria as previously described [13]. The diagnosis of AD cases was based on the presence of neurofibrillary tangles and neuritic plaques in the hippocampal formation and neocortical areas and met “intermediate” or “high” levels of AD Neuropathological Change according to the criteria of the National Institute on Aging﻿–Alzheimer’s Association (NIA–AA). DLB was defined as dementia occurring either at presentation or within one year of the onset of parkinsonism, with a brain distribution of α-synuclein pathology meeting DLB Consortium criteria for “intermediate” or “high” likelihood [67]. The Unified Staging System and McKeith criteria received good inter-rater reliability scores in a multi-center comprehensive analysis defining consensus criteria for the evaluation of Lewy body pathology in *post mortem* brains [8]. The diagnosis of VAD was based on the National Institute of Neurological Disorders and Stroke (NINDS) and the Association Internationale pour la Recherche et l’Enseignement en Neurosciences (AIREN) criteria [92].

Brain sections of the posterior superior temporal gyrus (Brodmann area 22) from ten AD cases, ten DLB cases, ten VAD cases and thirty age-matched non-demented subjects were evaluated for deposition of different post-translationally modified Aβ variants by immunohistochemistry. According to their respective Thal phase, the thirty age-matched non-demented subjects were further subdivided into control (Co; Thal 0–1) and pre-symptomatic AD (Pre-AD; Thal 2–5). Biochemical analyses were performed in unfixed brain tissue from the same location (Brodmann area 22) of the other brain hemisphere of the same cases. For detailed characterization of the cases, see Table 1.

**Table 1:** Human brain tissue of the posterior superior temporal gyrus used for immunohistochemical labelings and immunoassay analyses

Case #	Age [years]	PMI [h]	Gender *f* / *m*	Brain weight [g]	CAA yes / no	Braak score		Thal phase	APOE genotype	MMSE score	CERAD criteria	NIA-AA criteria	Unified LB stage
Co													
Co1	88	3	f	1030	no	II		1	3/4	n.a	not AD	not met	0
Co2	82	2.16	m	1160	yes	III		1	3/3	28	not AD	not met	0
Co3	91	1.5	m	1440	no	II		0	3/3	27	not AD	not met	0
Co4	53	3.66	m	1456	no	I		0	3/3	n.a	not AD	not met	0
Co5	61	2.33	m	1220	yes	I		1	3/3	n.a	not AD	not met	0
Co6	59	3.15	f	1162	no	I		1	3/3	n.a	not AD	not met	0
Co7	91	2.5	f	1185	yes	III		1	2/2	27	not AD	not met	0
Co8	74	3.25	m	1440	no	I		0	2/3	n.a	not AD	not met	0
Co9	82	2.07	f	1249	yes	I		1	2/3	29	not met	not met	0
Co10	90	5.42	m	1239	no	III		0	3/3	22	not met	not met	0
Co11	79	4.3	m	1156	no	II		0	2/3	28	not met	not met	0
Co12	99	3.5	f	975	no	III		0	3/3	29	not AD	not met	0
Co13	90	3.41	m	1150	no	I		1	3/3	27	not met	not met	0
Co14	87	3.08	f	1001	no	III		0	3/4	28	not met	not met	0
Co15	87	4	f	1196	yes	III		1	3/4	28	not met	not met	0
Co16	72	4.6	m	1262	yes	I		0	3/3	29	not met	not met	0
Co17	71	3.5	m	1344	no	III		0	3/3	27	not met	not met	0
Co18	100	3.87	m	1274	no	III		0	n. a	20	not met	not met	0
Co19	76	2.5	m	1340	no	I		0	3/3	n.a	not AD	not met	0
Co20	79	3	m	1100	no	I		0	3/3	n.a	not AD	not met	0
**Mean**	**80.55**	**3.24**	**7 / 13**	**1218.95**	**6 / 20**	**II**		**0**		**26.85**			**0**
Pre-AD													
Pre-AD1	81	2.75	m	1190	yes	III		5	3/3	25	not AD	not met	0
Pre-AD2	90	3	f	1150	no	III		3	3/3	27	not AD	not met	0
Pre-AD3	86	3	m	1055	no	I		2	3/3	n.a	not AD	not met	0
Pre-AD4	95	2.66	f	1140	no	III		2	3/3	n.a	not AD	not met	0
Pre-AD5	73	2.5	m	1240	no	II		2	2/3	30	not AD	not met	0
Pre-AD6	87	4	f	1145	no	III		2	3/3	n.a	not AD	not met	0
Pre-AD7	53	3.95	f	1015	no	II		2	3/3	n.a	not met	low	0
Pre-AD8	81	3	f	1275	yes	II		3	4/4	n.a	not AD	not met	0
Pre-AD9	79	2	m	1135	no	I		2	3/3	n.a	not AD	not met	0
Pre-AD10	70	2	f	1100	yes	I		2	4/4	n.a	not AD	not met	0
**Mean**	**79.50**	**2.89**	**6 / 4**	**1144.5**	**3 / 10**	**II**		**2**		**27.33**			**0**
AD													
AD1	76	3.08	f	862	yes		V	n.a	3/3	6	definite AD	high	0
AD2	92	3.73	f	1110	yes		VI	5	3/4	7	definite AD	high	0
AD3	75	3.83	m	1070	yes		V	5	3/4	3	definite AD	high	0
AD4	77	3.62	m	1180	yes		VI	5	3/4	10	definite AD	high	0
AD5	83	4	m	1184	yes		V	5	3/4	14	definite AD	high	0
AD6	83	3.62	f	1052	yes		V	5	3/3	17	definite AD	high	0
AD7	84	3.33	f	1142	yes		VI	5	3/3	24	definite AD	high	0
AD8	83	3.58	m	1105	yes		V	5	2/4	3	definite AD	high	0
AD9	75	2.97	m	1251	yes		V	5	3/4	25	definite AD	high	0
AD10	82	4	f	1098	yes		VI	5	3/3	19	definite AD	high	0
**Mean**	**81.00**	**3.58**	**5 / 5**	**1105.4**	**10 / 10**		**V**	**5**		**12.80**			**0**
DLB													
DLB1	78	3.33	m	1300	no		I	3	3/3	n.a	probable AD	intermediate	IV
DLB2	80	2.33	f	1080	no		III	4	3/4	0	probable AD	intermediate	IV
DLB3	87	3.75	m	1160	no		II	0	2/3	22	not AD	not met	III
DLB4	74	2.42	m	1422	no		II	3	3/3	9	definite AD	intermediate	IV
DLB5	97	3.47	f	1066	no		IV	0	3/3	27	not met	not met	IV
DLB6	68	2.5	m	1350	no		I	3	3/4	26	definite AD	intermediate	IV
DLB7	96	3.16	f	1195	no		III	0	3/3	n.a	not AD	not AD	III
DLB8	92	5	m	1200	yes		IV	4	3/4	20	probable AD	intermediate	IIb
DLB9	78	3	m	1225	no		I	5	2/3	n.a	probable AD	intermediate	IV
DLB10	74	3.33	m	1250	yes		III	2	3/3	16	not AD	low	IV
**Mean**	**82.40**	**3.23**	**3 / 7**	**1224.8**	**2 / 10**		**II**	**2**		**17.14**			**IV**
VAD													
VAD1	86	3	m	1120	no		III	0	3/3	20	not AD	not met	0
VAD2	106	2.25	f	896	yes		IV	0	2/3	19	not met	not met	0
VAD3	89	2.83	m	1158	no		III	0	3/3	27	not met	not met	0
VAD4	85	3	f	1080	no		I	n.a	3/3	20	not AD	not AD	0
VAD5	86	2.97	f	1082	no		IV	0	3/3	12	not met	not met	0
VAD6	92	3.32	f	1070	no		IV	0	3/3	27	not met	not met	0
VAD7	93	2.92	m	1015	no		II	3	2/3	19	definite AD	low	0
VAD8	92	2.63	f	1075	no		IV	0	3/3	28	not met	not met	0
VAD9	88	2	f	1170	yes		III	2	3/4	24	possible AD	low	0
VAD10	82	3.25	m	1290	no		III	0	3/4	2	not AD	not AD	0
**Mean**	**89.90**	**2.82**	**6 / 4**	**1095.6**	**2 / 10**		**III**	**1**		**19.80**			**0**

Bold represents mean values of the disease conditions

*PMI* post mortem interval; *f* female; *m* male; *CAA* Cerebral Amyloid Angiopathy; *Braak score* Braak neurofibrillary stage I–VI [18]; *Thal phase* Thal amyloid staging with phases 0 (A0), 1 (A1), 2 (A1), 3 (A2), 4 (A3) and 5 (A3) [112, 113]; *APOE genotype* apolipoprotein E genotype; *MMSE score* last Mini Mental State Examination score before death; *CERAD criteria* diagnosis of AD based on criteria of the Consortium to establish a registry for AD; *NIA-AA criteria* diagnosis of AD based on criteria of the National Institute on Aging–﻿Alzheimer’s Association; *Unified LB stage* unified staging system for Lewy body disorders with stage 0 (no Lewy bodies), I (olfactory bulb only), IIa (brainstem predominant), IIb (limbic predominant), III (brainstem and limbic), IV (neocortical) [12]

### Antibodies

For immunohistochemical labelings and immunoassay analyses, primary antibodies specific for the respective Aβ variants were used (for details, see Table 2). Antibodies raised against Aβ (3D6; epitope Aβ amino acids 1 to 5; parent antibody of Bapineuzumab), pGlu3-Aβ (J8), isoAsp7-Aβ (K11), pGlu11-Aβ (K13), 3NTyr10-Aβ (4C3) and isoAsp27-Aβ (F2) were generated by Fraunhofer IZI-MWT (Halle, Germany) (see Supplementary Information). The specificities of the antibodies 3D6 against Aβ [32, 38], J8 against pGlu3-Aβ [45], K11 against isoAsp7-Aβ [38], 1E4E11 against pSer8-Aβ [56], K13 against pGlu11-Aβ [52] and 5H11C10 against pSer26-Aβ [59] were recently demonstrated. For the characterization of the binding capacity and specificity of the newly generated monoclonal antibodies 4C3 raised against 3NTyr10-Aβ and F2 against isoAsp27-Aβ, immunoassays based on different N- or C-terminally truncated Aβ species as well as wild type Aβ were established (see Suppl. Figure 1 and 2). For the detection of Aβ(4-X), the polyclonal rabbit antibody 58–1 was used for immunohistochemical labeling [10] and the monoclonal antibody 18H6 was employed for immunoassay analyses [22]. In immunoassay analyses, the commercially available horseradish peroxidase (HRP)-conjugated antibody 4G8 (Biolegend, San Diego, CA, USA) against Aβ(17–24) and HRP-conjugated antibody Aβx-40 (clone 11A50B10; Biolegend) against Aβ(x-40) have been used for the detection of Aβ and Aβ40 species, respectively.

**Table 2:** Primary and secondary antibodies for detection of Aβ variants by immunohistochemistry (IHC) and immunoassay analyses

IHC and Immunoassay			IHC		Immunoassay
Antibody (Clone; Concentration [mg/ml])	Company / Supplier	Host	Pre-treatment	Antibody Dilution	Standard Curve Range or Concentration [ng/ml]
Aβ (3D6; 5.00)	Fraunhofer IZI (Halle, Germany)	Mouse	–	1:4000	0.39–0.006
pGlu3-Aβ (J8; 1.00)	Fraunhofer IZI (Halle, Germany)	Mouse	basic	1:1000	0.39–0.006
Aβ(4-X) (58–1; 1.00)	University Medical Center Göttingen (Göttingen, Germany)	Rabbit	–	1:5000	–
Aβ(4-X) (18H6; 0.60)	NYU Grossman School of Medicine (New York, USA)	Mouse	–	–	30–0.0073
isoAsp7-Aβ (K11; 3.00)	Fraunhofer IZI (Halle, Germany)	Mouse	–	1:6000	0.5–0.008
pSer8-Aβ (1E4E11; 1.00)	University Hospital Bonn (Bonn, Germany)	Mouse	acidic	1:1000	3.125–0.049
pGlu11-Aβ (K13; 2.50)	Fraunhofer IZI (Halle, Germany)	Mouse	–	1:1000	–
3NTyr10-Aβ (4C3; 1.00)	Fraunhofer IZI (Halle, Germany)	Mouse	–	1:400	6.25–0.01
pSer26-Aβ (5H11C10; 1.00)	University Hospital Bonn (Bonn, Germany)	Mouse	acidic	1:2000	2.187–0.003
isoAsp27-Aβ (F2; 1.64)	Fraunhofer IZI (Halle, Germany)	Mouse	–	1:6000	100–1.56
biotinylated donkey anti-mouse (-; 0.55)	Dianova, BIOZOL Diagnostica Vertrieb GmbH, (Eching, Germany)	Donkey	–	1:1000	–
biotinylated donkey anti-rabbit (-; 0.50)	Dianova, BIOZOL Diagnostica Vertrieb GmbH, (Eching, Germany)	Donkey	–	1:1000	–
HRP-conjugated 4G8 (4G8; 0.50)	Biolegend (San Diego, CA, USA)	Mouse	–	–	1000
HRP-conjugated Aβx-40 (11A50B10; 0.50)	Biolegend (San Diego, CA, USA)	Mouse	–	–	1000

Pre-treatments: basic: Tris-buffer (0.05 M; pH 8.0); acidic: citric acid/sodium citrate (0.1 M; pH 6.0) and 88% FA (v/v)

### Peptide synthesis

Peptides listed in Suppl. Table 1 were purchased from peptides & elephants GmbH (Hennigsdorf, Germany), BioCat GmbH (Heidelberg, Germany) or Peptide Specialty Laboratories GmbH (Heidelberg, Germany). Generation of full-length and N- or C-terminally truncated pGlu3-Aβ, 3NTyr10-Aβ and isoAsp27-Aβ peptides was performed by Fraunhofer IZI as previously described by Piechotta et al. [80] and Gnoth et al. [38].

### Immunohistochemical analysis

#### Single labeling immunohistochemistry

All immunohistochemical labelings on human brain tissue were performed on 40 µm thick free-floating brain sections at room temperature, unless stated otherwise. After optional pre-treatment (for pre-treatments, see Table 2), brain sections were washed in PBS containing 0.02% Tween 20 (PBS-T) and treated with 1% H_2_O_2_ in 60% methanol for 60 min. Unspecific staining was blocked by incubating brain sections in blocking solution (PBS-T with 2% (*w/v*) bovine serum albumin, 0.3% (*w/v*) milk powder, 0.5% (*v/v*) normal donkey serum) for 60 min before incubating brain sections with the primary antibodies in blocking solution at 4 °C for 42 h (for antibody concentrations, see Table 2). Brain sections were incubated in a 1:2 mixture of blocking solution and PBS-T containing the biotinylated donkey anti-mouse or anti-rabbit, respectively, secondary antibody (1:1000; Dianova, BIOZOL Diagnostica Vertrieb GmbH, Eching, Germany) for 60 min followed by incubation with ExtrAvidin-conjugated peroxidase (1:2000; Sigma, Merck KGaA, Darmstadt, Germany) in PBS-T for 60 min. Bound peroxidase was visualized in a solution containing 2–4 mg DAB, 40 mg ammonium nickel(II) sulfate and 5 µl H_2_O_2_ per 10 ml Tris-buffer (0.05 M; pH 8.0) yielding black epitope staining.

#### Light microscopy

Immunohistochemically stained human brain sections were digitized with an Axio-Scan.Z1 slide scanner connected with a LED light source and a Hitachi HV-F202SCL camera (Carl Zeiss AG, Oberkochen, Germany). Using a 20 × objective lens with 0.8 numerical aperture (Carl Zeiss AG), high-resolution images from areas of interest were taken. Images were analyzed using the Zeiss ZEN 3.8 imaging tool. Photoshop CS2 (Adobe Systems, San José, CA, USA) was used to process the images. Care was taken to apply the same brightness, sharpness, color saturation and contrast adjustments in the processing of the various pictures.

#### Automated image analysis

To quantify the stained plaque area of immunohistochemically labeled brain sections, Zeiss arivis cloud and Zeiss ZEN 3.8 Intellesis software were used for automated image analysis (for details see Suppl. Figure 3). For each Aβ variant and for all 60 cases, three representative regions of interest (ROIs) with an area of 4 mm^2^ each were chosen of the grey matter spanning all cortical layers. A separate training was conducted for each Aβ variant staining using Zeiss arivis cloud. After several iterating training cycles to enhance the capability of the machine learning algorithm to detect immunohistochemically stained plaques, all 60 cases were analyzed by the trained algorithm in a batch analysis using Zeiss ZEN Intellesis software. Obviously false positive detection of unspecific staining was manually removed upon visual inspection.

#### Staging of plaque morphology and vascular deposition

The staging of compact, coarse-grained, cored and diffuse plaques stained for a specific Aβ variant was performed on digitized human brain slices with Zeiss ZEN 3.8 according to Boon et al. [15]. The plaque load of each plaque morphology was ranked between no staining ( – ), some positive staining (+  – ), positive staining ( +) and prominent positive staining with high plaque load (+ +). Vascular Aβ deposition was evaluated similarly and differentiated between 0% ( – ), 1–40% (+  – ), 41–80% ( +) and 81–100% (+ +) of cases with vascular deposition per clinical group.

### Immunoassays

#### Human brain tissue preparation

Tissue blocks of human temporal cortex were prepared in the frontal plane according to the atlas of the human brain and stored at – 80 °C until usage. To prepare human brain for Aβ immunoassay analysis, the protocol of Gnoth et al. [38] was followed with adaptations. In short, a sample of about 500 mg temporal cortex was homogenized in TBS buffer (20 mM Tris/HCl, 150 mM NaCl, 5 mM KCl, pH 7.5) supplemented with Protease Inhibitor Cocktail Tablets (Roche Diagnostics GmbH, Mannheim, Germany) and 1 mM 4-(2-Aminoethyl)-benzolsulfonylfluorid Hydrochlorid (Carl Roth GmbH + Co. KG, Karlsruhe, Germany) at a concentration of 200 mg/ml by using a Precellys homogenizer (VWR International GmbH, Darmstadt, Germany), followed by sonication for 10 s. After centrifugation of the homogenate at 100,000xg for 1 h, supernatant (TBS fraction) was obtained. The resulting pellet was dissolved to 250 mg/ml in 5 M GdmCl, followed by an incubation step in an overhead shaker at room temperature for 3 h and a subsequent centrifugation step at 100,000xg for 1 h. Again, supernatant (GdmCl fraction) was collected and the pellet was resuspended in 500 µl 70% FA, followed by sonication for 20 s and neutralization by 2.9 ml 3.5 M Tris (FA fraction).

#### Quantification of Aβ variants using immunoassay analysis

The performance of Aβ and isoAsp7-Aβ immunoassay analyses was previously described by Gnoth et al. [38]. In short, coating antibodies were immobilized on polystyrene 96-well microtiter plates at 4 °C overnight. After blocking (4 °C, 2 h), sample dilutions (TBS fraction 1:2–1:50, GdmCl fraction 1:40–1:5000, FA fraction 1:100–1:5000) were selected according to subsequent adsorption signals within the linear range of the standard curve. For the standard curve, synthetic standard peptides were serially diluted and added to the wells in duplicate. This was followed by an incubation period at 4 °C for 2 h. For detection of bound Aβ peptides, the HRP-conjugated antibody 4G8 was used to detect Aβ, pGlu3-, isoAsp7-, pSer8- and 3NTyr10-Aβ. The HRP-conjugated antibody Aβx-40 was used for pSer26- and isoAsp27-Aβ detection. Both detection antibodies were diluted to a final concentration of 1 μg/ml (see Table 2), added to the samples and incubated at 4 °C for 1 h. A color reaction with commercially available HRP substrate 3,3′,5,5′-3,3,5,5-Tetramethylbenzidine (TMB; SureBlue Reserve TMB Microwell Peroxidase Substrate (1-component); KPL, LGC Clinical Diagnostics, Milford, MA, USA) was performed and stopped by the addition of 1.2 N H_2_SO_4_. Absorption at 450/540 nm was determined by a Tecan Sunrise plate reader (Tecan Group Ltd., Männedorf, Switzerland). The standard curve was calculated from measured absorption by a 4-Parameter-Logistic-Fit: *y* = A2 + (A1 − A2)/(1 + (*x*/ × 0)^p). Novel immunoassays were adapted and specified in the same way (see Table 2). For specification purposes, the stability of the antibodies in the matrix as well as LOD and LOQ were determined.

Quantification of the Aβ(4-X) variant was carried out as electrochemiluminescence assay (Meso Scale Discovery, MSD, Rockville, MD, USA). Biotinylated 18H6 ([22]; 0.6 µg/ml), as well as sulfo-tagged 4G8 (1:50; Meso Scale Discovery, MSD) were used as capture and detection antibodies, respectively, on MSD GOLD 96-well Small Spot Streptavidin Plates (Meso Scale Discovery, MSD) following the manufacturer protocol for the human Aβ antibody set. Standard curves were prepared using synthetic Aβ(4–40) peptides and data analysis including standard curve calculations was carried out with the Discovery Workbench 4.0.12 software package (Meso Scale Discovery, MSD).

### Thioflavin T fibril formation assays

Aβ aggregation studies were based on Piechotta et al. [80]. Lyophilized Aβ peptides were dissolved in 1,1,1,3,3,3-hexafluoro-2-isopropanol (HFIP) to obtain homogeneous preparations without seeds. For peptide preparation, the HFIP was evaporated and immediately prior to analysis, peptide pellets were dissolved in 20 µl of 0.1 M NaOH and incubated for 10 min. After adding 380 µl PBS (138 mM NaCl, 8 mM Na_2_HPO_4_, 1.5 mM KH_2_PO_4_, 3 mM KCl, pH 7.1), neutralization with 0.1 M HCl followed. All work steps were carried out on ice. Peptide concentration was determined by Pierce BCA Protein Assay Kit (Thermo Fisher Scientific, Waltham, MA, USA) and adjusted to 10 µM or 15 µM. To monitor the fibril formation of Aβ variants, monomeric Aβ peptide was co-incubated with 20 µM thioflavin T (ThT) in 96-well microtiter plates (PS, half area, black µClear; Greiner Bio-One, Kremsmünster, Austria). Measurements were carried out in triplicates. The plate was sealed with adhesive film and incubated at 37 °C and 300 rpm for 20 h. ThT fluorescence intensity was measured using a CLARIOstar (BMG Labtech, Ortenberg, Germany) plate reader (excitation 440 nm, emission 490 nm) and normalized to the initial ThT fluorescence signals. Kinetic parameters of the ThT curves of the fibril-forming Aβ variants were calculated using logistic sigmoid functions in GraphPad PRISM (version 10, San Diego, CA, USA).

### Transmission electron microscopy

Transmission electron microscopy (TEM) of Aβ aggregates was based on Köppen et al. [53]. In short, fibril samples (10 µl) from an aggregation reaction as described above were directly incubated on an EM Tec Formvar Carbon TEM Support Film on nickel 200 square mesh (Micro to Nano, Haarlem, Netherlands) at room temperature for 10 min and washed three times with distilled water. Staining was obtained with 2% (v/v) uranyl acetate (SERVA Electrophoresis GmbH, Heidelberg, Germany) for 1 min. Fibrils were imaged with a LEO EM 912 Omega TEM (Carl Zeiss AG) at 80 kV, and digital micrographs were obtained with a dual-speed 2 K-on-axis CCD camera-based YAG scintillator and the software ImageSP (version 1.2.13.17 TRS-Tröndle, Moorenweis, Germany).

### Statistical analysis of immunohistochemical and immunoassay data

For statistical analysis, the mean plaque area and the mean amount of Aβ variant was used. Using a Python script for immunohistochemical data, the total plaque area of one ROI of one case and one Aβ variant was calculated relative to the total area of the ROI (4 mm^2^). The mean plaque area was then calculated from the total plaque area of three ROIs per case. The mean amount of Aβ variant per case was calculated using two technical immunoassay replicates. In the case of the Aβ(4-X) electrochemiluminescence assay, a single measurement was performed. Determination of statistical significance of differences of the stained plaque areas and the amount of a specific Aβ variant, respectively, between disease conditions was conducted with One-Way ANOVAs followed by Tukey’s multiple comparisons test using GraphPad PRISM. Differences between groups were considered statistically significant for *p* values < 0.05. Correlations of plaque area or amount of Aβ variants relative to Braak stage, Thal phase and MMSE were calculated as Pearson Correlations using GraphPad PRISM. The Pearson correlation coefficient r and the P value (two-tailed, confidence interval 95%) are indicated in each plot. Pearson correlation was considered to be moderate with correlation coefficients of 0.40 ≤ r < 0.70, as strong with 0.70 ≤ r < 0.90 and as very strong with 0.90 ≤ r ≤ 1.00. Correlations with 0.00 ≤ r < 0.40 were interpreted as weak and negligible [105]. Heat Maps show the mean plaque areas, the mean Aβ variant amounts and the Pearson correlation coefficient r of the immunohistochemical or biochemical correlations, respectively (values are not normalized).

## Results

### Immunohistochemical analyses

**Fig. 3:**
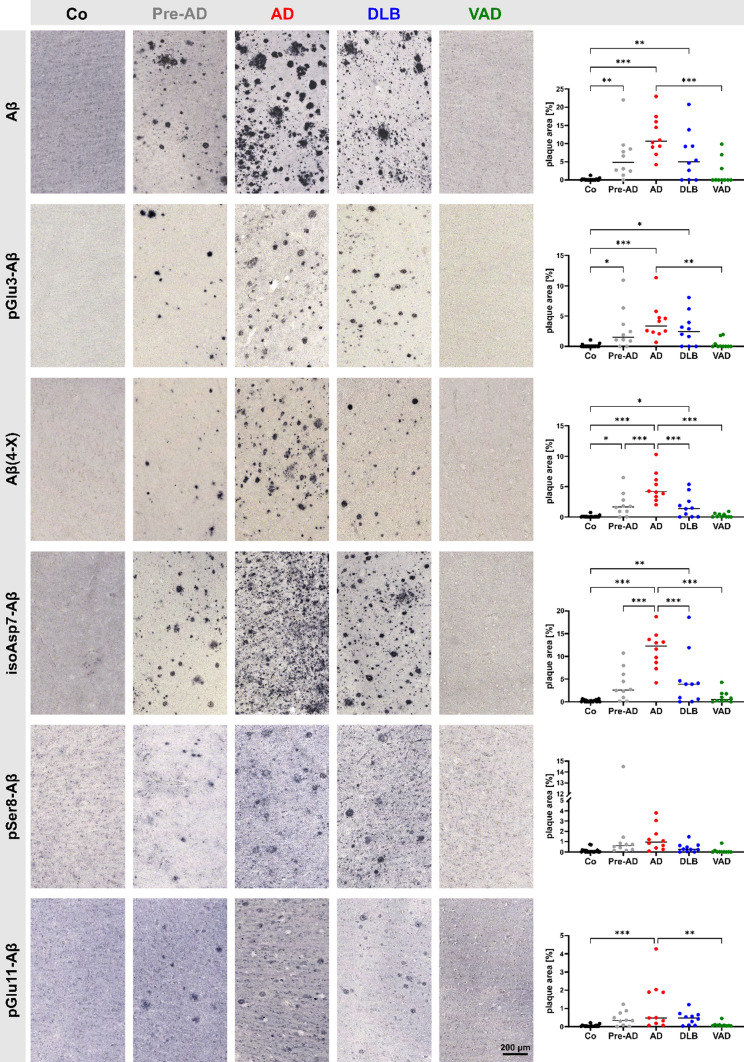
Representative examples of immunohistochemical labeling of human cortical brain tissue from control cases and different clinical conditions using antibodies to detect specific post-translational Aβ modifications as indicated (left). Respective quantifications of Aβ plaque load as a percentage of brain area covered by plaques is presented (right). Note that the Y-axes differ between the individual Aβ variants. Differences between clinical groups are statistically significant at *p < 0.05; **p < 0.01; ***p < 0.001. Medians are indicated by horizontal lines

First, the abundance of post-translationally modified Aβ peptides in amyloid plaques was quantified. The Aβ variants 3NTyr10-Aβ, pSer26-Aβ and isoAsp27-Aβ were not detected in amyloid plaque-like formations and, thus, excluded from this analysis. The immunohistochemical appearance of these Aβ variants is documented in Suppl. Figure 4. All other Aβ variants were, in varying quantities, detected in amyloid plaques (Fig. 3). In control cases, Braak stage I–III and Thal phase 0–1, a very minor plaque load of < 1% of the grey matter brain area was calculated for all Aβ peptide variants (Fig. 3). In the Pre-AD cases, Braak stage I–III and Thal phase 2–5, the median plaque load varied between 4.8% for Aβ and 0.3% for pGlu11-Aβ. Among the remaining Aβ PTMs, the isoAsp7-Aβ variant was most abundant (2.6% plaque load) followed by Aβ(4-X) (1.7% plaque load), pGlu3-Aβ (1.5% plaque load) and pSer8-Aβ (0.6% plaque load) (Fig. 3). Among all clinical conditions, the highest Aβ plaque load was detected for AD, Braak stage V–VI and Thal phase 5 (Fig. 3). For the different Aβ variants, median plaque load values between 10.7% for Aβ and 0.5% for pGlu11-Aβ were detected. The abundance of isoAsp7-Aβ (12.3% plaque load) was similar to that of Aβ and was followed by Aβ(4-X) (4.2% plaque load), pGlu3-Aβ (3.4% plaque load) and pSer8-Aβ (1.0% plaque load). Also in DLB, Braak stage I to IV and Thal phase 0–5, the isoAsp7-Aβ was most abundant among all Aβ variants (3.9% plaque load), followed by pGlu3-Aβ (2.5% plaque load), Aβ(4-X) (1.4% plaque load) and pGlu11-Aβ and pSer8-Aβ with less than 1% plaque load each (Fig. 3). In the VAD cases, Braak stage I–IV and Thal phase 0–3, all Aβ variants accounted for less than 1% plaque load, each.

Together, the immunohistochemical analyses of plaque load revealed the highest abundance of all Aβ variants analyzed in AD, followed by DLB, Pre-AD and VAD, the latter two being close to control cases. Regarding the different Aβ variants, isoAsp7-Aβ plaque load was most abundant, followed by Aβ(4-X) and pGlu3-Aβ at similar levels and pGlu11-Aβ and pSer8-Aβ with the lowest plaque occupancy.

**Fig. 4:**
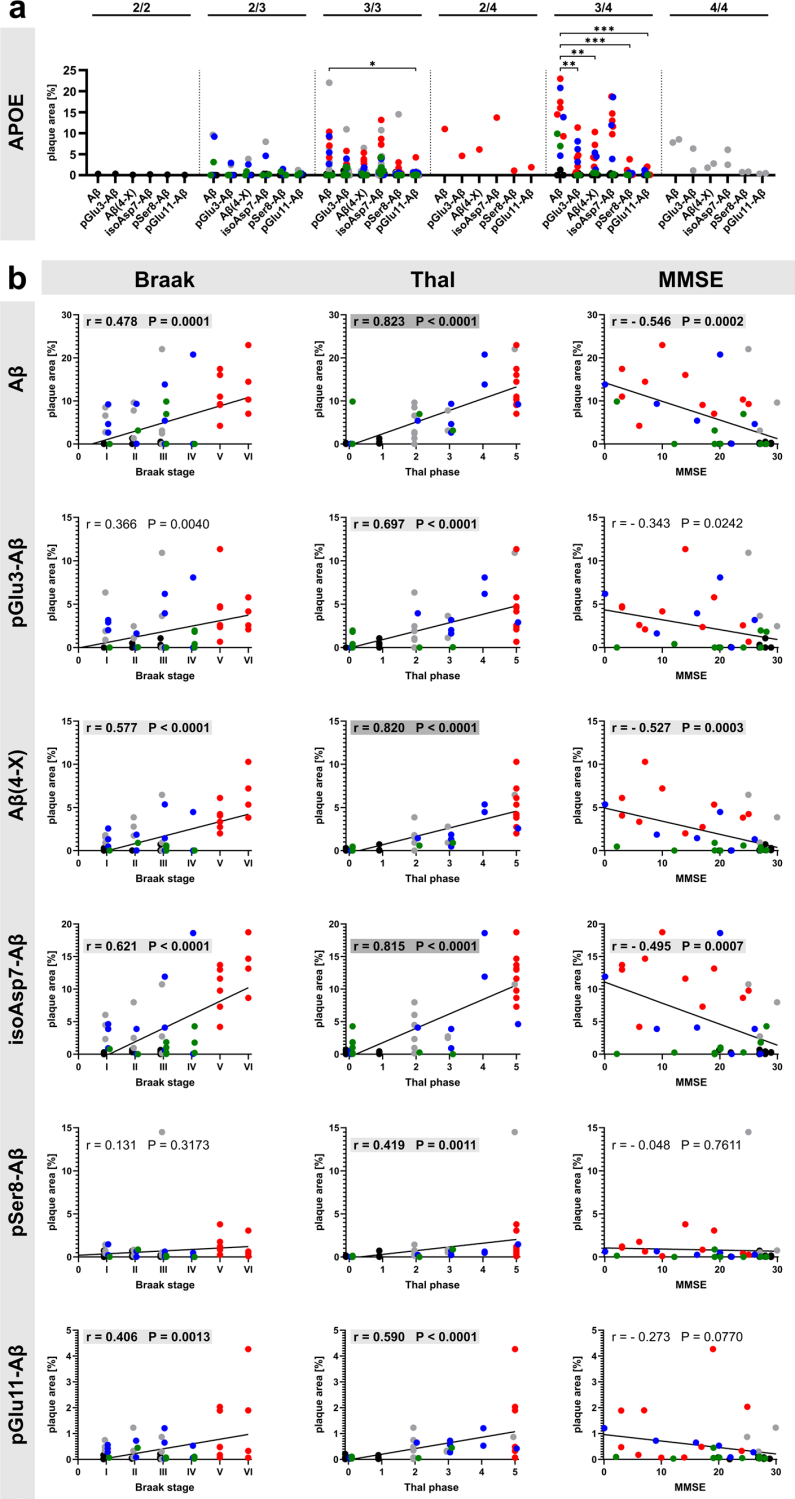
**a** Subgroup analyses of the abundance of Aβ variants by APOE genotype. Note the high plaque load for all Aβ variants in APOE 3/4 carriers and the particularly high abundance of isoAsp7-Aβ compared to the other Aβ PTMs in this subgroup. **b** Pearson correlation analyses between the immunohistochemically quantified plaque load for individual Aβ variants and the neuropathological Braak Tau stages (column 1) and Thal amyloid phases (column 2). In addition, correlations between the respective abundance of Aβ variants and the MMSE scores (column 3) are presented. Pearson correlation was considered as moderate with correlation coefficients of 0.40 ≤ r < 0.70 (highlighted in light grey) and as strong with 0.70 ≤ r < 0.90 (highlighted in grey). Note the moderate and strong correlations of isoAsp7-Aβ with Braak Tau stages, Thal amyloid phases and MMSE scores. The individual cases relate to the clinical condition as follows: black—Co; grey—Pre-AD; red—AD; blue—DLB; green—VAD

We next asked whether there is an association of the Aβ load with APOE genotype and whether there might be correlations between the abundance of the Aβ variants investigated and the histopathological Braak and Thal staging on one hand and the MMSE score on the other. The subgroup sizes defined by APOE genotype differed markedly with *N* = 1 for the 2/2, *N* = 8 for the 2/3, *N* = 1 for the 2/4, *N* = 34 for the 3/3, *N* = 13 for the 3/4 and *N* = 2 for the 4/4 allele (see Table 1). The highest abundance for all Aβ variants was detected in APOE 3/4 carriers, followed by APOE 3/3 carriers (Fig. 4a). Within the APOE 3/4 subgroup, the mean plaque load for all Aβ PTMs was significantly lower than that of Aβ, except for isoAsp7-Aβ, which displayed similar levels. As expected, the Aβ variants showed a higher correlation with Thal amyloid phases than with Braak Tau stages (Fig. 4b). The strongest correlations with Thal phases were observed for Aβ, Aβ(4-X) and isoAsp7-Aβ (*r* values 0.70 ≤ *r* < 0.90 indicate a strong correlation) and were highlighted in grey. Moderate correlations (*r* values 0.40 ≤ *r* < 0.70) were marked with a light grey. Regarding the MMSE, we observed moderate negative correlations for Aβ, Aβ(4-X) and isoAsp7-Aβ (Fig. 4b).

**Fig. 5:**
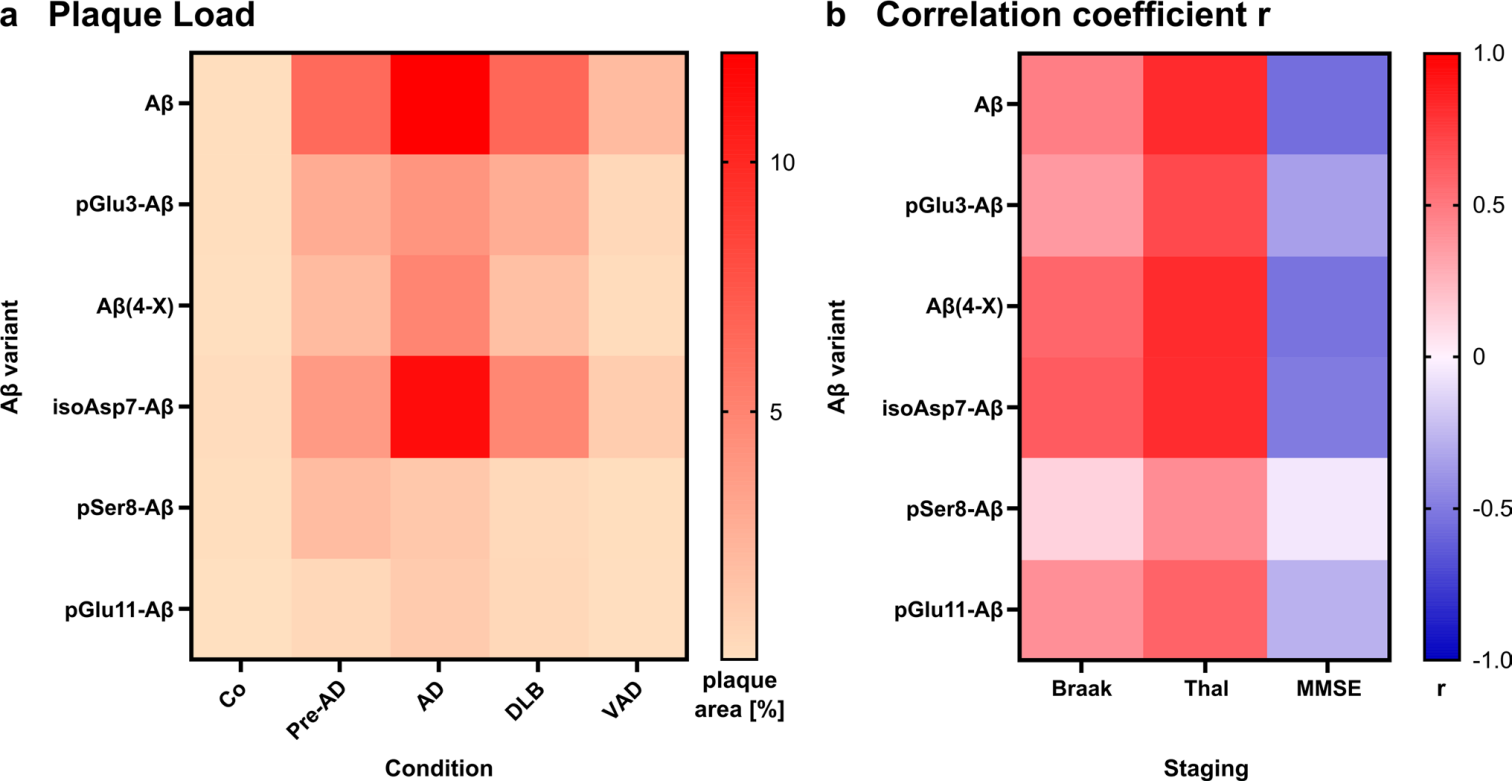
Heat maps for the plaque load of Aβ variants and the correlation coefficient r of the Pearson correlation analyses. **a** Immunohistochemically quantified plaque load of Aβ variants is depicted for the different conditions. **b** For all correlations between the plaque load of Aβ variants and Braak and Thal staging and MMSE score, the correlation coefficient r as a measure for the quality of the correlation is presented. Note that the typical correlation pattern is not observed for pSer8-Aβ

To better visualize the plaque load for the individual Aβ variants in the different types of dementia and the strength of correlations with Braak and Thal staging and MMSE scores, respectively, heat maps were established (Fig. 5). These indicate the particularly high abundance of isoAsp7-Aβ in Pre-AD, AD and DLB (Fig. 5a), as well as a positive correlation between isoAsp7-Aβ plaque load and histopathological Braak and Thal staging (Fig. 5b). In addition, a moderate correlation of isoAsp7-Aβ and Aβ(4-X) with the decline in MMSE is evident (Fig. 5b).

**Fig. 6:**
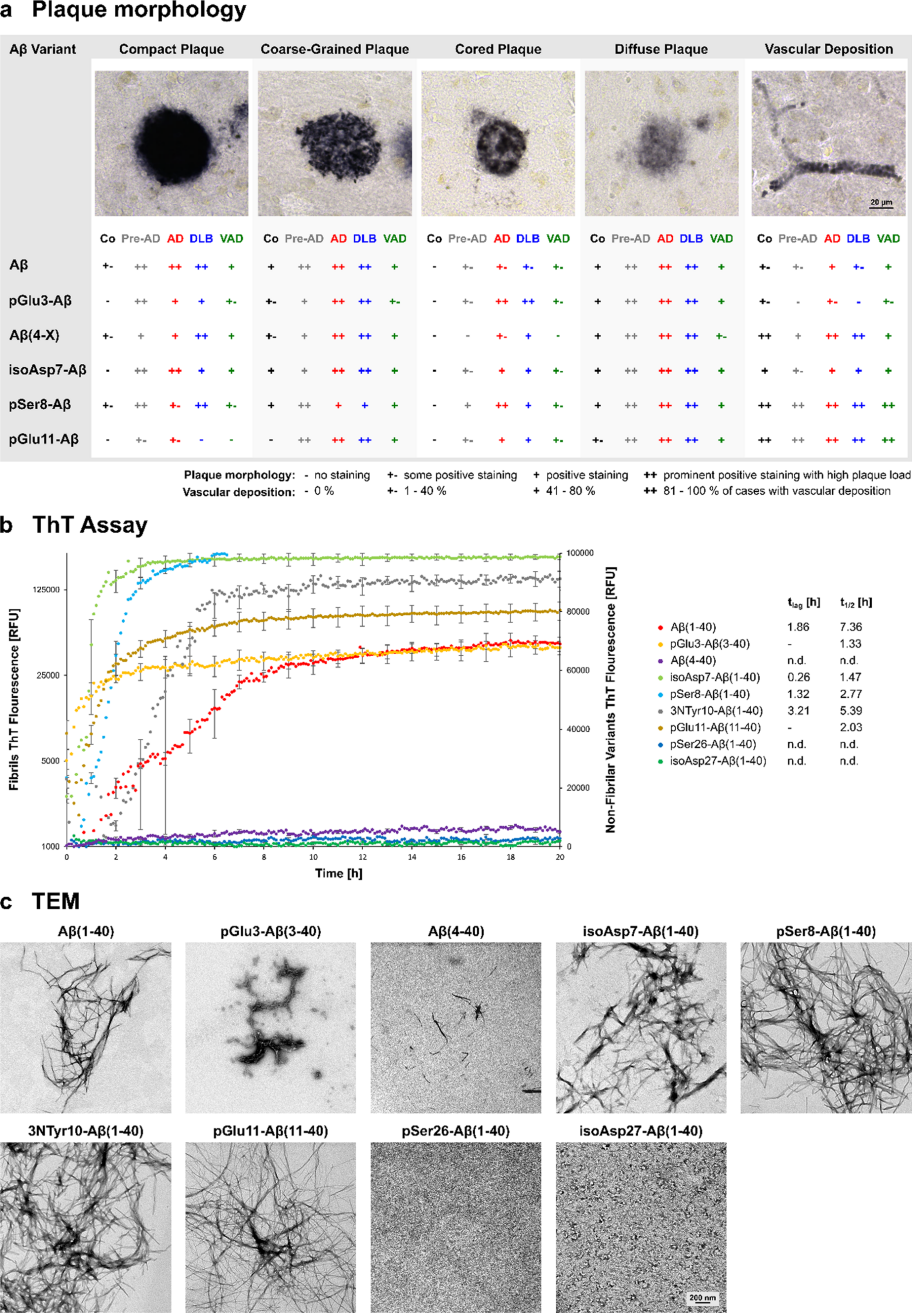
**a** Association of Aβ, pGlu3-Aβ, Aβ(4-X), isoAsp7-Aβ, pSer8-Aβ and pGlu11-Aβ variants with amyloid plaques of compact, coarse-grained, cored and diffuse morphologies as well as with cerebral vessels. The images on the top show labeling of Aβ. The quantifications below indicate a high abundance for most Aβ variants in all plaque types, in particular in AD and DLB. In blood vessels, pSer8-Aβ and pGlu11-Aβ variants were particularly abundant in all clinical conditions, whereas pGlu3-Aβ and isoAsp7-Aβ were present in a smaller subset of patients in all clinical conditions. **b** Aggregation curves of time-dependent fibril formation of the Aβ variants. Note the instant fibril formation of pGlu3-Aβ(1–40). As compared to unmodified Aβ(1–40), the variants pGlu3-Aβ(1–40), isoAsp7-Aβ(1–40), pSer8-Aβ(1–40) and 3NTyr10-Aβ(1–40) showed shorter lag phases, indicating more rapid formation of β-sheet containing aggregates. We did not observe significant fibril formation of Aβ(4–40), pSer26-Aβ(1–40) and isoAsp27-Aβ(1–40) variants. t_lag_—lag phase, t_1/2_—half maximum ThT fluorescence intensity time. **c** Electron microscopic images of fibrils derived from the respective Aβ variants

We also wanted to reveal whether specific Aβ variants are associated with amyloid plaque types of distinct morphology and with cerebral blood vessels. Therefore, we graded the abundance of each Aβ variant separately according to its respective detection in the following categories of plaques: compact, coarse-grained, cored and diffuse, as well as in blood vessels. This was done for all clinical conditions and cases (Fig. 6a). The staging was made according to Boon et al. [15] and differentiated between no staining ( – ), some positive staining (+  – ), positive staining ( +) and prominent positive staining with high plaque load (+ +). AD and DLB cases displayed a high abundance of all plaque types labeled for all Aβ variants. Diffuse plaques were strongly labeled by all Aβ antibodies and were also highly abundant in the Pre-AD cases. Regarding the prominent labeling of isoAsp7-Aβ and Aβ(4-X) variants, isoAsp7-Aβ was found to be more abundant in plaques of compact and of cored morphology than Aβ(4-X) (Fig. 6a). For the staining of cerebral blood vessels, the grading differentiated between no vascular amyloid deposits in any case of a clinical group ( – ), in 1 to 40% of the cases (+  – ), in 41–80% of the cases ( +) and in 81–100% of the cases (+ +). The pSer8-Aβ and pGlu11-Aβ variants were highly abundant in blood vessels in all clinical conditions including Pre-AD and control cases (Fig. 6a). In comparison, the Aβ(4-X) variant was less abundant in the vasculature of the VAD and Pre-AD group, whereas pGlu3-Aβ and isoAsp7-Aβ were present in all clinical conditions in a smaller subset of patients.

We next asked whether the Aβ variants present in amyloid plaques display different aggregation kinetics than their counterparts not detected in plaques. ThT assays were employed to monitor the time-dependent fibril formation of all Aβ(x-40) variants. Intriguingly, the pSer26-Aβ(1–40) and isoAsp27-Aβ(1–40) variants which had not been localized in plaques only showed low ThT fluorescence. On the contrary, 3NTyr10-Aβ(1–40) which had not been detected in amyloid plaques, did show ThT fluorescence and, thus, robust time-dependent fibril formation in the aggregation assay. The other Aβ variants detected in amyloid plaques also showed ThT fluorescence with instant fibril formation for pGlu3-Aβ(1–40), isoAsp7-Aβ(1–40) and pGlu11-Aβ(1–40), and lag phases ranging from 1.32 h for pSer8-Aβ(1–40) to 3.21 h for 3NTyr10-Aβ(1–40) (Fig. 6b). The Aβ(4-X) variant is an exception from this pattern, since it was highly abundant in amyloid plaques, but the Aβ(4–40) variant did not form fibrils under the conditions of the ThT assay. This might indicate that the Aβ(4–42) variant dominates in plaques.

To reveal specific characteristics of the fibrils derived from distinct Aβ variants, TEM was performed (Fig. 6c). In general, the TEM images confirmed and substantiated the data of the fibrillation curves obtained in the ThT assay, although a few Aβ(4–40) fibrils were detected (Fig. 6c). Remarkably, pGlu3-Aβ(1–40) and isoAsp7-Aβ(1–40) fibrils appeared to be shorter and thicker than fibrils of the other Aβ variants (Fig. 6c).

### Biochemical analyses

We used immunoassay analyses to quantify those Aβ variants that were shown above to be present in amyloid plaques. The Aβ variants 3NTyr10-Aβ, pSer26-Aβ and isoAsp27-Aβ were not detected in amyloid plaque-like formations and, thus, immunoassay analyses of these Aβ variants are shown in Suppl. Figure 5.

**Fig. 7:**
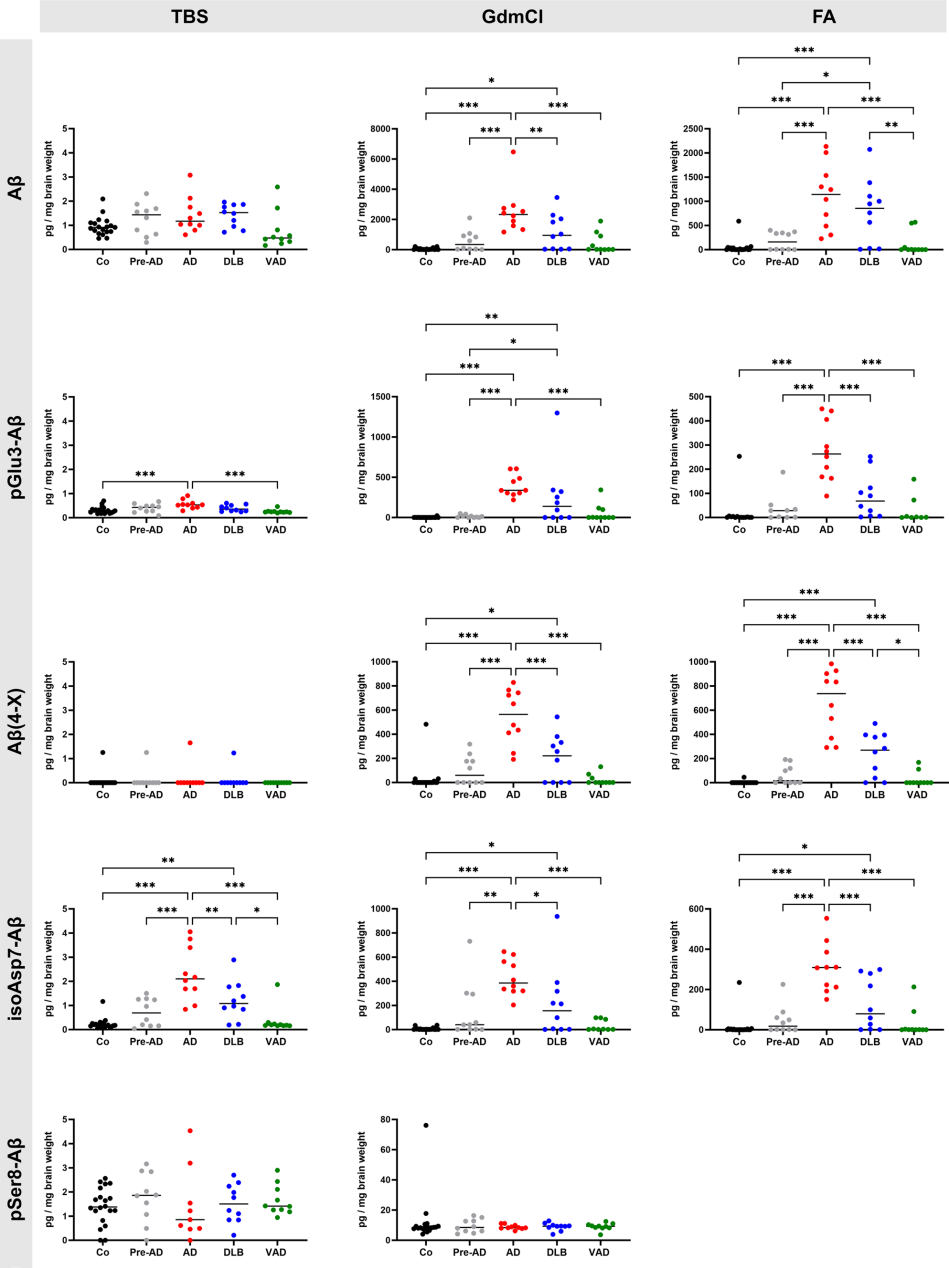
Quantification of Aβ variants by immunoassays. Note that the Y-axes differ for the individual Aβ variants and for TBS, GdmCl and FA fractions. Differences between clinical groups are statistically significant at *p < 0.05; **p < 0.01; ***p < 0.001. Medians are indicated by horizontal lines

Aβ was separated based on solubility in TBS, GdmCl and FA fractions. For each Aβ variant, less than 1% of the sum of all fractions was detected in the TBS fraction, whereas GdmCl and FA fractions accounted for about 50% of each Aβ variant (Fig. 7). Due to potential dephosphorylation of pSer8-Aβ in FA [21], pSer8-Aβ was not measured in this fraction.

Consistent with immunohistochemical data on the quantification of the respective plaque load, immunoassay analyses revealed the highest Aβ concentrations in AD, followed by DLB, Pre-AD, VAD and controls. Specifically, in control cases, Braak stage I to III and Thal phase 0–1, a low amount of all Aβ variants was measured (Fig. 7). In the Pre-AD cases, Braak stage I to III and Thal phase 2–5, the median amount of Aβ was close to control subjects and varied between 341 pg/mg for Aβ and 9 pg/mg for pSer8-Aβ in GdmCl and between 161 pg/mg for Aβ and 17 pg/mg for Aβ(4-X) in FA.

In AD cases, the highest Aβ concentration was measured in the GdmCl and the FA fraction for Aβ with 2,331 pg/mg and 1,141 pg/mg, respectively. The lowest concentration was measured for pSer8-Aβ with 8 pg/mg in GdmCl. In GdmCl fractions, the amount of Aβ(4-X) (564 pg/mg) was highest among the Aβ variants, followed by isoAsp7-Aβ (386 pg/mg) and pGlu3-Aβ (337 pg/mg). In FA, the same pattern as in GdmCl was observed with the highest concentration of Aβ(4-X) (737 pg/mg), followed by isoAsp7-Aβ (309 pg/mg) and pGlu3-Aβ (263 pg/mg).

In DLB cases, Braak stage I to IV and Thal phase 0–5, Aβ amounts in the GdmCl fraction ranged from 9 pg/mg for pSer8-Aβ to 951 pg/mg for Aβ. The variants pGlu3-Aβ, Aβ(4-X) and isoAsp7-Aβ showed similar concentrations with around 69 to 269 pg/mg in both GdmCl and FA. In the VAD cases, Braak stage I to IV and Thal phase 0–3, all Aβ variants accounted for low concentrations in GdmCl and FA.

**Fig. 8:**
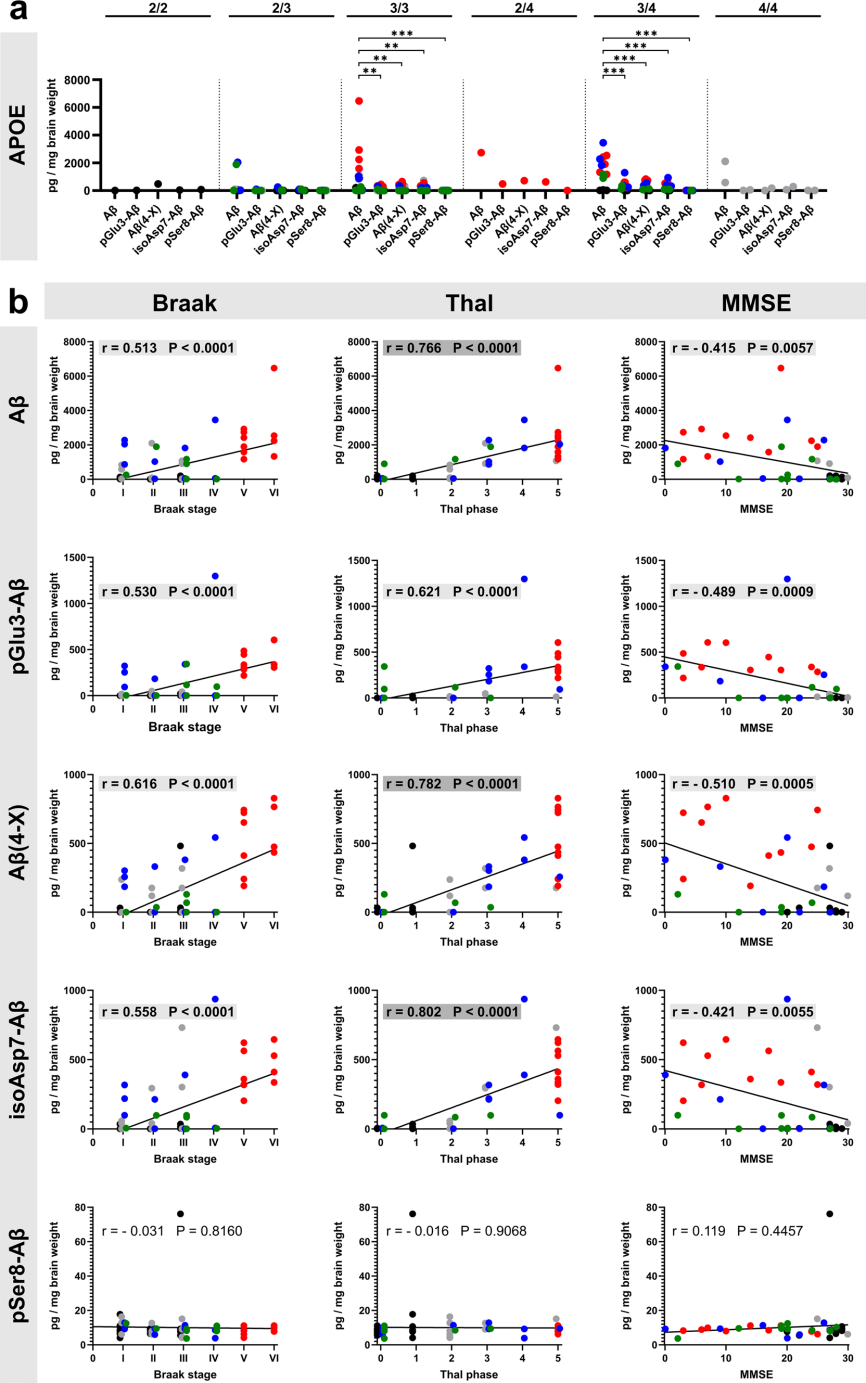
**a** Subgroup analyses of the abundance of Aβ variants by APOE genotype in GdmCl fractions. Note the high quantity of Aβ in APOE 3/3 and APOE 3/4 carriers. **b** Pearson correlation analyses between the load for individual Aβ variants quantified by immunoassays in the GdmCl fractions and the neuropathological Braak Tau stages (column 1) and Thal amyloid phases (column 2). In addition, correlations between the abundance of Aβ variants and the MMSE scores (column 3) are presented. Pearson correlation was considered as moderate with correlation coefficients of 0.40 ≤ r < 0.70 (highlighted in light grey) and as strong with 0.70 ≤ r < 0.90 (highlighted in grey). The individual cases relate to the clinical condition as follows: black—Co; grey—Pre-AD; red—AD; blue—DLB; green – VAD

In analogy to the correlations of immunohistochemically determined plaque load, we performed a subgroup analysis of the Aβ load by APOE genotype and correlated the abundance of the Aβ variants quantified by immunoassay with Braak and Thal staging and with MMSE score (Fig. 8). The highest quantities for all Aβ variants were detected in APOE 3/4 carriers, followed by APOE 3/3 carriers (Fig. 8a). In these APOE genotypes, Aβ PTMs displayed significantly lower concentrations than Aβ. In addition, the Aβ variants showed a higher correlation with Thal amyloid phases than with Braak Tau stages, with the strongest correlations for Aβ, Aβ(4-X) and isoAsp7-Aβ (r values between 0.70 and 0.90 indicate a strong correlation; highlighted in grey), and a moderate correlation for pGlu3-Aβ (r value 0.621; marked with a light grey) (Fig. 8b). Regarding the MMSE, we observed moderate negative correlations with *r *values between – 0.415 and – 0.510 for all Aβ variants except for pSer8-Aβ (Fig. 8b).

The correlation analyses of the biochemically determined concentrations of the Aβ variants with histopathological and clinical data revealed results almost identical to the immunohistochemical data, indicating strong inter-assay reliability of our analytical methods.

**Fig. 9:**
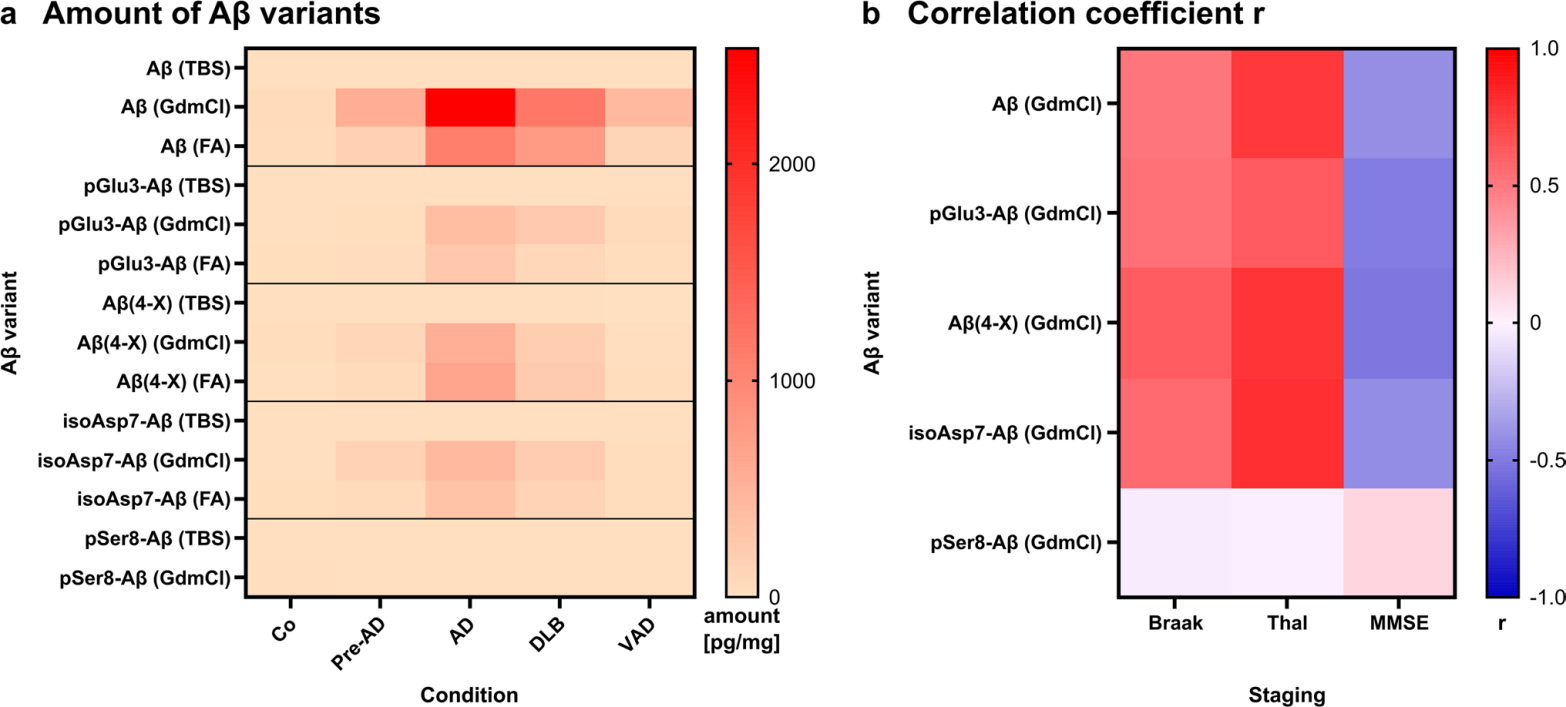
Heat maps for the amount of Aβ variants quantified in the TBS, GdmCl and FA fractions and the correlation coefficient r of the Pearson correlation analyses. **a** Aβ variants quantified by immunoassays are depicted for the different clinical conditions. **b** For all correlations between the Aβ concentrations and Braak and Thal staging and MMSE score, the correlation coefficient r as a measure for the quality of the correlation is presented. Note that the typical correlation pattern is not observed for pSer8-Aβ

To better visualize the abundance of the individual Aβ variants in different tissue fractions and in distinct types of dementia and to indicate the strength of correlations with Braak and Thal staging and MMSE scores, heat maps were established (Fig. 9). These indicate the particular high abundance of pGlu3-Aβ, Aβ(4-X) and isoAsp7-Aβ in GdmCl and FA fractions in AD and to a lesser extent in DLB (Fig. 9a). For all Aβ variants except pSer8-Aβ, a moderate to strong correlation to the Thal amyloid phases was observed (Fig. 9b). Likewise, for all Aβ variants, except pSer8-Aβ, moderate negative correlations with MMSE were observed (Fig. 9b).

## Discussion

In this study, we present an extensive and, to our knowledge, the most comprehensive analysis of eight distinct Aβ PTMs in human brain tissue affected by different types of dementia. Our key discovery is that the isoAsp7 modification is a highly abundant Aβ PTM in all clinical conditions including Pre-AD, AD, DLB and VAD.

### Association of Aβ variants with amyloid plaques in different types of dementia

The two Aβ variants with the highest abundance identified in this study in all clinical conditions were the isoAsp7-Aβ and Aβ(4-X) variants (see Suppl. Figure 6).

The isoAsp7-Aβ variant appeared to be very prominent among all clinical conditions observed when quantifying immunohistochemical plaque load and concentrations of Aβ variants in GdmCl and FA fractions by immunoassay. We observed strong correlations of the abundance of this variant with Thal phase and moderate correlations with the decline in MMSE. In addition, we report robust and instant fibril formation without lag phase in aggregation assays. This Aβ modification was identified over 30 years ago in the brain parenchyma [90]. The isoAsp7-Aβ variant was observed to induce cerebral amyloidosis when administered peripherally to transgenic mice with amyloid pathology [54] and to be more neurotoxic towards human neural stem cells than Aβ(1–42) [69]. In addition, an Aβ variant with a dual isoAsp modification at residues 1 and 7 was shown to be abundant in parenchymal and vascular Aβ deposits in AD [75], as well as in the Iowa variant of familial AD [115]. Another study showed the neuroprotective effect of protein-L-isoaspartyl methyltransferase, an ubiquitous enzyme that catalyses the conversion of isoAsp back to Asp, against Aβ oligomers by increasing the size and reducing both hydrophobicity and toxicity of Aβ oligomers [23]. We observed an instant fibril formation for the isoAsp7-Aβ variant in the ThT assay. Intriguingly, targeting isoAsp7-Aβ with the monoclonal antibody K11 used in this study has already been demonstrated to reduce amyloid pathology and to ameliorate behavioural deficits in 5xFAD mice [38, 39]. This makes this Aβ variant particularly interesting as a therapeutic target for different types of dementia.

Aβ(4-X) was identified in AD brain and has been shown to account for more than 60% of the FA extractable Aβ peptides from amyloid plaque cores that were accessible to Edman protein sequencing [66, 68]. However, considering the abundance of pGlu3-Aβ and pGlu11-Aβ that escape Edman protein sequencing, its proportion in plaques should be lower but still significant. Subsequently, its presence was demonstrated by mass spectrometric analysis in the AD brain [83], in parenchymal and vascular deposits in the Iowa variant of familial AD [115], as well as in Danish amyloid (ADan) and Aβ co-deposits in Familial Danish Dementia [114]. Recently, Aβ(4-X) was shown to be produced by ADAMTS4, a secreted metalloprotease that is exclusively expressed in oligodendrocytes [120]. Moreover, N-terminal Aβ truncation at position 4 was demonstrated to result in the formation of poorly soluble, aggregation-prone peptides with high amyloidogenic propensity and the potential of exacerbated fibrillary deposit formation [22]. A study using a polyclonal Aβ(4-X) antibody confirmed the presence of Aβ(4-X) in amyloid plaques in two transgenic AD mouse models but also showed that Aβ(4-X) was present in human AD cases in blood vessels and in neuritic plaques but not in diffuse amyloid deposits [123]. These findings were further expanded by a study using another monoclonal antibody (clone 18H6) against Aβ(4-X). In this study, an association between Aβ(4-X) and cored plaques, CAA and also diffuse plaques was reported in human AD brain tissue, along with an enhanced brain retention of oligomeric Aβ(4-X) [94]. In our study, the polyclonal Aβ(4-X) antibody detected the Aβ variant in all plaque types as well as in vascular amyloid deposits which is in line with the initial characterization of this antibody [10] and a recent study using the monoclonal antibody 18H6 against Aβ(4-X) [125]. In our ThT assay and transmission electron microscopy, only little fibril formation was detected for Aβ(4–40), suggesting that the majority of Aβ(4-X) deposited in brain parenchyma and cerebral blood vessels is Aβ(4–42).

There are a number of reports in the literature that point to the presence of individual or multiple Aβ variants in brain tissue of one or more clinical conditions. For example, Aβ, the pGlu3-Aβ and pSer8-Aβ variants were compared side-by-side using immunohistochemical and Western blot analyses in control, Pre-AD and AD [89]. This allowed for a biochemical staging of Aβ deposition, where both Aβ PTMs were absent in stage 1 as the earliest preclinical stage, only pGlu3-Aβ was present in stage 2 and both modified Aβ variants were present in the last stage of Aβ aggregation, stage 3 [89]. The higher abundance of pGlu3-Aβ compared to pSer8-Aβ in Pre-AD (median plaque load of 1.5% for pGlu3-Aβ and 0.6% for pSer8-Aβ) and AD cases (median plaque load of 3.4% for pGlu3-Aβ and 1.0% for pSer8-Aβ) was also demonstrated in our study and was quantified in detail by automated immunohistochemical analysis and immunoassay methods. Another study analyzed the pGlu3-Aβ and pSer8-Aβ variants in cerebral amyloid angiopathy (CAA) and found a similar pattern of vascular amyloid stages with pGlu3-Aβ preceding pSer8-Aβ deposits in microvessels [36]. It should be noted that the appearance of Aβ coincides with widespread Tau pathology at later stages of AD and that there is Tau co-pathology of DLB [70, 106]. Although Tau pathology was not investigated in the present study, we would like to point out that Tau is an important mediator required for pGlu3-Aβ toxicity [77]. Moreover, pGlu3-Aβ load in AD predicted the hyperphosphorylated Tau load and was related to the severity of AD neuropathology and clinical dementia [65]. In addition, pGlu3-Aβ and isoAsp-Aβ displayed low immunoreactivity in non-demented controls and were significantly increased in AD [75].

Other studies focused on pSer8-Aβ and pGlu3-Aβ, individually, in different clinical conditions and related animal models. For example pSer8-Aβ was identified in AD brain and reported to form neurotoxic oligomers that act as nuclei for Aβ fibril formation [55, 56]. In addition, pSer8-Aβ was found to be present in Down syndrome and transgenic mouse models with Aβ pathology [60] and in brains of non-human primates and canines [58]. However, the pSer8-Aβ variant was demonstrated to be exclusively associated with a subset of Aβ plaques and vascular deposits in familial and sporadic AD and to be absent or only detectable at very small amounts in control, DLB and VAD brains [7]. This accumulation pattern of the phosphorylated Aβ variant pSer8-Aβ in Pre-AD, AD, DLB and VAD brain tissue was substantiated in our present study.

The presence of the pGlu3-Aβ variant in AD amyloid plaques was initially reported by the Saido and Roher groups [63, 97]. It was subsequently reported to be present in the brains of humans, non-human primates, canines and transgenic mice with amyloid pathology [30, 44, 71, 72, 122]. Because of its instant aggregation [104], high neurotoxicity [2, 77], compromised degradation [95, 96] and seeding capacity [77, 101], pGlu3-Aβ emerged as a pharmacologic target for AD therapy. There are two strategies of interfering with pGlu3-Aβ, (i) prevention of its generation by inhibition of glutaminyl cyclase [46, 103] and (ii) specific targeting of existing pGlu3-Aβ by immunotherapy [24, 26, 27, 29, 31]. Recently, intravenous infusions with Donanemab (Kisunla) targeting pGlu3-Aβ have been approved by the U.S. Food and Drug Administration for early symptomatic AD treatment [51].

As discussed above, in the course of AD, pGlu3-Aβ is present earlier than the pSer8-Aβ variant and its abundance is higher. In addition, compared to the other Aβ variants, pGlu3-Aβ displayed lower concentrations than isoAsp7-Aβ and Aβ(4-X) in brains from Pre-AD, AD, DLB and VAD cases. The related pGlu11-Aβ variant, on the other hand, has been studied less intensively, but was shown to be co-localized with pGlu3-Aβ in amyloid plaques in AD brain tissue and to form the central plaque core [79, 111]. In addition, structural characteristics and neurotoxicity of pGlu11-Aβ were recently reported [98, 99]. We here observed that the pGlu11-Aβ variant only accounts for a small proportion of the Aβ plaque load in all clinical conditions investigated.

Another important aspect is that longer Aβ42 and Aβ43 peptides are more directly linked to AD based on early pathology, familial AD mutations in presenilins, Down syndrome amyloid pathology and Aβ biomarkers than the shorter Aβ40 variants [16, 33, 73, 85]. This also holds true for higher neurotoxicity and faster aggregation characteristics of Aβ42 versus Aβ40 [48, 49, 81]. In the present study, pGlu3-Aβ(3–40), isoAsp7-Aβ(1–40), pGlu11-Aβ(11–40) and pSer8-Aβ(1–40) showed faster aggregation compared to unmodified Aβ(1–40). Thus, these PTMs could increase the pathogenicity of Aβ40 variants towards that of Aβ42.

### Aβ variants not associated with amyloid plaques

In the present study, the Aβ variants 3NTyr10-Aβ, pSer26-Aβ and isoAsp27-Aβ were not detected in amyloid plaques and only the 3NTyr10-Aβ(1–40) variant was shown to form fibrils in aggregation experiments of Aβ(1–40) variants in vitro. Importantly, in the case of immunohistochemical labeling and immunoassay analyses, pre-analytical treatments of brain tissue and samples may influence the final results and should be considered when comparing different results. However, it seemed striking that the plaque-associated Aβ variants investigated were post-translationally modified at the N-terminus of the Aβ peptide. Due to the high β-sheet content of aggregated Aβ in plaques [3], it might be difficult for monoclonal antibodies against pSer26-Aβ and isoAsp27-Aβ to bind to their epitopes, which might be sterically inaccessible in the plaque-like formation.

For 3NTyr10-Aβ, the NOS2-catalyzed nitration was shown by using enzyme inhibitors and knock-out mice [61]. This Aβ variant was prone to fibril formation and was detected in the core of amyloid plaques in brains from AD patients and transgenic mice with amyloid pathology [61]. Since these initial data were obtained with a polyclonal antiserum, we developed a specific monoclonal antibody targeting this particular Aβ variant for the present study. While we could reproduce the biochemical characteristics of 3NTyr10-Aβ in the ThT and TEM assays (see Fig. 6b, c), we did not detect an association of 3NTyr10-Aβ with amyloid plaques in any of the clinical conditions (see Suppl. Figure 4). Instead, the monoclonal antibody showed an intraneuronal localization of 3NTyr10-Aβ. Discrepancies in the staining pattern of the polyclonal antiserum and the monoclonal antibody might arise from impacts of Aβ conformation and secondary structure or different detection sensitivities of the antibodies as well as brain tissue storage conditions and pre-analytical sample preparations.

The pSer26-Aβ has recently been demonstrated to give rise to stable oligomeric assemblies with high neurotoxicity. Furthermore, the pSer26 modification has been shown to impair the fibrillation of the Aβ peptide [57, 88]. In a transgenic AD mouse model and in human AD brain, pSer26-Aβ was found to be localized particularly in intraneuronal deposits [57]. The lack of fibril formation of pSer26-Aβ (see Fig. 6) and its intraneuronal labeling was confirmed here in all types of dementia under investigation, especially in AD and DLB (see Suppl. Figure 4).

In addition to the previously described isoAsp7-Aβ variant (see above), we here also aimed at an analysis on the potential formation of isoAsp27-Aβ, for which we generated and characterized a monoclonal antibody (see Suppl. Figure 2). There are several reports in the literature showing that Asp isomerization may be a phenomenon associated with aging and neurodegeneration in general and in AD in particular [47, 74, 78, 93]. For this reason, antibodies raised against isoAsp-modified Aβ have been proposed as indicators of the plaque age [28]. In general, isoAsp is formed at sites with Asn or Asp amino acid precursors, whereas the rate of isoAsp formation from Asn precursors is typically one-to-two orders of magnitude faster than from Asp [35, 41]. Hence, it is tempting to speculate that isoAsp27-Aβ might be formed in addition to isoAsp7-Aβ. However, the formation and deposition of isoAsp27-Aβ appears negligible and to be restricted to intracellular compartments. A potential rationale for this observation might be the placement of Asn27 at the contact area of Aβ protofilaments to form fibrils [124]. A modification of this residue to isoAsp might prevent the formation of fibrils. Likewise, isoAsp27-Aβ(1–40) did not show fibril formation in our analysis.

Taken together, although not abundantly present in different types of dementia, the 3NTyr10-Aβ, pSer26-Aβ and isoAsp27-Aβ variants were detected in the soma of pyramidal neurons. Hence, a potential influence on neuronal and synaptic dysfunction cannot be excluded, as previously suggested for pSer26-Aβ [57].

It is quite obvious that substantial modifications to the majority of Aβ peptides are restricted to the N-terminal residues 1–8, as also suggested recently [76]. Cryo-EM and NMR data collectively suggest that these residues are not incorporated into fibrils and are therefore structurally flexible and accessible to enzyme catalysis or spontaneous isoAsp formation.

### Association of Aβ variants with cerebral blood vessels

The pGlu3-Aβ and isoAsp7-Aβ variants were observed to be less frequently associated with cerebral microvessels than the other Aβ variants investigated in this study. However, both Aβ variants, as well as the pGlu11-Aβ, are not completely absent from blood vessels, a finding also reported recently for AD [109]. Vascular Aβ deposits are believed to contribute to blood–brain-barrier disruption and to account for amyloid-related imaging abnormalities (ARIAs), edema and hemorrhages, during the anti-Aβ immunotherapies [11, 43, 50]. Thus, targeting Aβ variants less frequently present in blood vessels should be expected to reduce these adverse events. However, in the TRAILBLAZER trials using the antibody Donanemab to target the pGlu3-Aβ variant in patients, such events were not less frequent than in other clinical trials [43, 108].

## Conclusions

Post-translationally modified Aβ peptides play an important role in the initiation of aggregation processes that result in the formation of oligomeric/ fibrillary assemblies with high neurotoxicity. We hypothesized that different types of dementia are characterized by specific patterns of modified Aβ variants. Surprisingly, we did not identify such a disease-specific signature of post-translationally modified Aβ peptides but a rather consistent pattern of Aβ variants across different clinical entities. We discovered isoAsp7-Aβ as the most abundant Aβ variant, followed by Aβ(4-X) and pGlu3-Aβ, in all types of dementia assessed here. It remains an open question whether the most abundant Aβ variant is the best pharmacologic target. However, in preclinical studies, the efficacy of antibodies against isoAsp7-Aβ [38] and pGlu3-Aβ [27, 31] has already been demonstrated and the pGlu3-Aβ antibody Donanemab was recently approved for AD therapy. We believe that it is worth considering Donanemab treatment in other types of dementia and that isoAsp7-Aβ and Aβ(4-X) are additional targets for immunotherapy in different clinical conditions with an amyloid component.

## Supplementary Information

Below is the link to the electronic supplementary material.

Supplementary file1 (DOCX 32273 KB)

## Data Availability

The data sets used and analyzed during the current study are available from the corresponding author on reasonable request.

## Abbreviations

Aβ: Amyloid β
AD: Alzheimer’s disease
APOE: Apolipoprotein E
ARIA: Amyloid-related imaging abnormality
CAA: Cerebral amyloid angiopathy
DAB: 3,3’-Diaminobenzidine
DLB: Dementia with Lewy bodies
FA: Formic acid
GdmCl: Guanidinium chloride
HFIP: 1,1,1,3,3,3-Hexafluoro-2-isopropanol
HRP: Horseradish peroxidase
LB stage: Unified Staging System for Lewy Body Disorders
MMSE: Mini Mental State Examination
NOS2: Inducible nitric oxide synthase
PBS: Phosphate-buffered saline
PBS-T: PBS containing 0.02% Tween 20
PMI: *Post mortem* Interval
Pre-AD: Pre-symptomatic AD
PTM: Post-translational modifications
ROI: Region of interest
TBS: Tris-buffered saline
TEM: Transmission electron microscopy
ThT: Thioflavin T
TMB: 3,3′,5,5′-3,3,5,5-Tetramethylbenzidin
VAD: Vascular dementia

## References

[1] Abramov E, Dolev I, Fogel H, Ciccotosto GD, Ruff E, Slutsky I (2009) Amyloid-beta as a positive endogenous regulator of release probability at hippocampal synapses. Nat Neurosci 12:1567–1576. 10.1038/nn.2433 19935655 10.1038/nn.2433

[2] Alexandru A, Jagla W, Graubner S, Becker A, Bäuscher C, Kohlmann S et al (2011) Selective hippocampal neurodegeneration in transgenic mice expressing small amounts of truncated Aβ is induced by pyroglutamate-Aβ formation. J Neurosci 31:12790–12801. 10.1523/JNEUROSCI.1794-11.2011 21900558 10.1523/JNEUROSCI.1794-11.2011 PMC6623394

[3] Antzutkin ON, Leapman RD, Balbach JJ, Tycko R (2002) Supramolecular structural constraints on Alzheimer’s beta-amyloid fibrils from electron microscopy and solid-state nuclear magnetic resonance. Biochemistry 41:15436–15450. 10.1021/bi0204185 12484785 10.1021/bi0204185

[4] Arezoumandan S, Cousins KAQ, Ohm DT, Lowe M, Chen M, Gee J et al (2024) Tau maturation in the clinicopathological spectrum of Lewy body and Alzheimer’s disease. Ann Clin Transl Neurol 11:673–685. 10.1002/acn3.51988 38263854 10.1002/acn3.51988 PMC10963284

[5] Arima K, Uéda K, Sunohara N, Arakawa K, Hirai S, Nakamura M et al (1998) NACP/alpha-synuclein immunoreactivity in fibrillary components of neuronal and oligodendroglial cytoplasmic inclusions in the pontine nuclei in multiple system atrophy. Acta Neuropathol 96:439–444. 10.1007/s004010050917 9829806 10.1007/s004010050917

[6] Arima K, Hirai S, Sunohara N, Aoto K, Izumiyama Y, Uéda K et al (1999) Cellular co-localization of phosphorylated tau- and NACP/alpha-synuclein-epitopes in lewy bodies in sporadic Parkinson’s disease and in dementia with Lewy bodies. Brain Res 843:53–61. 10.1016/S0006-8993(99)01848-X 10528110 10.1016/s0006-8993(99)01848-x

[7] Ashby EL, Miners JS, Kumar S, Walter J, Love S, Kehoe PG (2015) Investigation of A β phosphorylated at serine 8 (pAβ) in Alzheimer’s disease, dementia with Lewy bodies and vascular dementia. Neuropathol Appl Neurobiol 41:428–444. 10.1111/nan.12212 25556395 10.1111/nan.12212

[8] Attems J, Toledo JB, Walker L, Gelpi E, Gentleman S, Halliday G et al (2021) Neuropathological consensus criteria for the evaluation of Lewy pathology in post-mortem brains: a multi-centre study. Acta Neuropathol 141:159–172. 10.1007/s00401-020-02255-2 33399945 10.1007/s00401-020-02255-2 PMC7847437

[9] Baba M, Nakajo S, Tu PH, Tomita T, Nakaya K, Lee VM et al (1998) Aggregation of alpha-synuclein in Lewy bodies of sporadic Parkinson’s disease and dementia with Lewy bodies. Am J Pathol 152:879–884 9546347 PMC1858234

[10] Bader AS, Gnädig MU, Fricke M, Büschgens L, Berger LJ, Klafki HW et al (2023) Brain region-specific differences in amyloid-β plaque composition in 5XFAD mice. Life (Basel) 13:1053. 10.3390/life13041053 37109582 10.3390/life13041053 PMC10145597

[11] Barakos J, Purcell D, Suhy J, Chalkias S, Burkett P, Marsica Grassi C et al (2022) Detection and management of amyloid-related imaging abnormalities in patients with Alzheimer’s disease treated with anti-amyloid beta therapy. J Prev Alzheimers Dis 9:211–220. 10.14283/jpad.2022.21 10.14283/jpad.2022.21 35542992

[12] Beach TG, Adler CH, Lue L, Sue LI, Bachalakuri J, Henry-Watson J et al (2009) Unified staging system for Lewy body disorders: correlation with nigrostriatal degeneration, cognitive impairment and motor dysfunction. Acta Neuropathol 117:613–634. 10.1007/s00401-009-0538-8 19399512 10.1007/s00401-009-0538-8 PMC2757320

[13] Beach TG, Adler CH, Sue LI, Serrano G, Shill HA, Walker DG et al (2015) Arizona study of aging and neurodegenerative disorders and brain and body donation program. Neuropathology 35:354–389. 10.1111/neup.12189 25619230 10.1111/neup.12189 PMC4593391

[14] Bluhm A, Schrempel S, Schilling S, von Hörsten S, Schulze A, Roßner S et al (2022) Immunohistochemical demonstration of the pGlu79 α-synuclein fragment in Alzheimer’s disease and its Tg2576 mouse model. Biomolecules 12:1006. 10.3390/biom12071006 35883562 10.3390/biom12071006 PMC9312983

[15] Boon BDC, Bulk M, Jonker AJ, Morrema THJ, van den Berg E, Popovic M et al (2020) The coarse-grained plaque: a divergent Aβ plaque-type in early-onset Alzheimer’s disease. Acta Neuropathol 140:811–830. 10.1007/s00401-020-02198-8 32926214 10.1007/s00401-020-02198-8 PMC7666300

[16] Borchelt DR, Thinakaran G, Eckman CB, Lee MK, Davenport F, Ratovitsky T et al (1996) Familial Alzheimer’s disease-linked presenilin 1 variants elevate Abeta1-42/1-40 ratio in vitro and in vivo. Neuron 17:1005–1013. 10.1016/s0896-6273(00)80230-5 8938131 10.1016/s0896-6273(00)80230-5

[17] Bouter Y, Dietrich K, Wittnam JL, Rezaei-Ghaleh N, Pillot T, Papot-Couturier S et al (2013) N-truncated amyloid β (Aβ) 4–42 forms stable aggregates and induces acute and long-lasting behavioral deficits. Acta Neuropathol 126:189–205. 10.1007/s00401-013-1129-2 23685882 10.1007/s00401-013-1129-2 PMC3722453

[18] Braak H, Braak E (1991) Neuropathological stageing of Alzheimer-related changes. Acta Neuropathol 82:239–259. 10.1007/BF00308809 1759558 10.1007/BF00308809

[19] Braak H, Braak E, Bohl J (1993) Staging of Alzheimer-related cortical destruction. Eur Neurol 33:403–408. 10.1159/000116984 8307060 10.1159/000116984

[20] Brody DL, Magnoni S, Schwetye KE, Spinner ML, Esparza TJ, Stocchetti N et al (2008) Amyloid-beta dynamics correlate with neurological status in the injured human brain. Science 321:1221–1224. 10.1126/science.1161591 18755980 10.1126/science.1161591 PMC2577829

[21] Bylund DB, Huang TS (1976) Decomposition of phosphoserine and phosphothreonine during acid hydrolysis. Anal Biochem 73:477–485. 10.1016/0003-2697(76)90197-4 962058 10.1016/0003-2697(76)90197-4

[22] Cabrera E, Mathews P, Mezhericher E, Beach TG, Deng J, Neubert TA et al (2018) Aβ truncated species: implications for brain clearance mechanisms and amyloid plaque deposition. Biochim Biophys Acta Mol Basis Dis 1864:208–225. 10.1016/j.bbadis.2017.07.005 28711595 10.1016/j.bbadis.2017.07.005 PMC5875988

[23] Chatterjee T, Das G, Chatterjee BK, Ghosh S, Chakrabarti P (2023) The role of protein-L-isoaspartyl methyltransferase (PIMT) in the suppression of toxicity of the oligomeric form of Aβ42, in addition to the inhibition of its fibrillization. ACS Chem Neurosci 14:2888–2901. 10.1021/acschemneuro.3c00281 37535852 10.1021/acschemneuro.3c00281

[24] Crehan H, Liu B, Kleinschmidt M, Rahfeld J-U, Le KX, Caldarone BJ et al (2020) Effector function of anti-pyroglutamate-3 Aβ antibodies affects cognitive benefit, glial activation and amyloid clearance in Alzheimer’s-like mice. Alzheimers Res Ther 12:12. 10.1186/s13195-019-0579-8 31931873 10.1186/s13195-019-0579-8 PMC6958628

[25] Crescenzi O, Tomaselli S, Guerrini R, Salvadori S, D’Ursi AM, Temussi PA et al (2002) Solution structure of the Alzheimer amyloid beta-peptide (1–42) in an apolar microenvironment. Similarity with a virus fusion domain. Eur J Biochem 269:5642–5648. 10.1046/j.1432-1033.2002.03271.x 12423364 10.1046/j.1432-1033.2002.03271.x

[26] Cynis H, Frost JL, Crehan H, Lemere CA (2016) Immunotherapy targeting pyroglutamate-3 Aβ: prospects and challenges. Mol Neurodegener 11:48. 10.1186/s13024-016-0115-2 27363697 10.1186/s13024-016-0115-2 PMC4929720

[27] DeMattos RB, Lu J, Tang Y, Racke MM, DeLong CA, Tzaferis JA et al (2012) A plaque-specific antibody clears existing β-amyloid plaques in Alzheimer’s disease mice. Neuron 76:908–920. 10.1016/j.neuron.2012.10.029 23217740 10.1016/j.neuron.2012.10.029

[28] Fonseca MI, Head E, Velazquez P, Cotman CW, Tenner AJ (1999) The presence of isoaspartic acid in beta-amyloid plaques indicates plaque age. Exp Neurol 157:277–288. 10.1006/exnr.1999.7058 10364440 10.1006/exnr.1999.7058

[29] Frost JL, Liu B, Kleinschmidt M, Schilling S, Demuth H-U, Lemere CA (2012) Passive immunization against pyroglutamate-3 amyloid-β reduces plaque burden in Alzheimer-like transgenic mice: a pilot study. Neurodegener Dis 10:265–270. 10.1159/000335913 22343072 10.1159/000335913 PMC3702016

[30] Frost JL, Le KX, Cynis H, Ekpo E, Kleinschmidt M, Palmour RM et al (2013) Pyroglutamate-3 amyloid-β deposition in the brains of humans, non-human primates, canines, and Alzheimer disease-like transgenic mouse models. Am J Pathol 183:369–381. 10.1016/j.ajpath.2013.05.005 23747948 10.1016/j.ajpath.2013.05.005 PMC3730768

[31] Frost JL, Liu B, Rahfeld J-U, Kleinschmidt M, O’Nuallain B, Le KX et al (2015) An anti-pyroglutamate-3 Aβ vaccine reduces plaques and improves cognition in APPswe/PS1ΔE9 mice. Neurobiol Aging 36:3187–3199. 10.1016/j.neurobiolaging.2015.08.021 26453001 10.1016/j.neurobiolaging.2015.08.021 PMC4641825

[32] Fuller JP, Stavenhagen JB, Christensen S, Kartberg F, Glennie MJ, Teeling JL (2015) Comparing the efficacy and neuroinflammatory potential of three anti-abeta antibodies. Acta Neuropathol 130:699–711. 10.1007/s00401-015-1484-2 26433971 10.1007/s00401-015-1484-2 PMC4612324

[33] Galasko D, Chang L, Motter R, Clark CM, Kaye J, Knopman D et al (1998) High cerebrospinal fluid tau and low amyloid beta42 levels in the clinical diagnosis of Alzheimer disease and relation to apolipoprotein E genotype. Arch Neurol 55:937–945. 10.1001/archneur.55.7.937 9678311 10.1001/archneur.55.7.937

[34] Galpern WR, Lang AE (2006) Interface between tauopathies and synucleinopathies: a tale of two proteins. Ann Neurol 59:449–458. 10.1002/ana.20819 16489609 10.1002/ana.20819

[35] Geiger T, Clarke S (1987) Deamidation, isomerization, and racemization at asparaginyl and aspartyl residues in peptides. Succinimide-linked reactions that contribute to protein degradation. J Biol Chem 262:785–794 3805008

[36] Gerth J, Kumar S, Rijal Upadhaya A, Ghebremedhin E, von Arnim CAF, Thal DR et al (2018) Modified amyloid variants in pathological subgroups of β-amyloidosis. Ann Clin Transl Neurol 5:815–831. 10.1002/acn3.577 30009199 10.1002/acn3.577 PMC6043770

[37] Glenner GG, Wong CW (1984) Alzheimer’s disease: initial report of the purification and characterization of a novel cerebrovascular amyloid protein. Biochem Biophys Res Commun 120:885–890. 10.1016/S0006-291X(84)80190-4 6375662 10.1016/s0006-291x(84)80190-4

[38] Gnoth K, Piechotta A, Kleinschmidt M, Konrath S, Schenk M, Taudte N et al (2020) Targeting isoaspartate-modified Aβ rescues behavioral deficits in transgenic mice with Alzheimer’s disease-like pathology. Alzheimers Res Ther 12:149. 10.1186/s13195-020-00719-x 33189132 10.1186/s13195-020-00719-x PMC7666770

[39] Gnoth K, Geissler S, Feldhaus J, Taudte N, Ilse V, Zürner S et al (2022) Evidence for enhanced efficacy of passive immunotherapy against beta-amyloid in CD33-negative 5xFAD mice. Biomolecules 12:399. 10.3390/biom12030399 35327591 10.3390/biom12030399 PMC8945487

[40] Gueorguieva I, Willis BA, Chua L, Chow K, Ernest CS, Wang J et al (2023) Donanemab exposure and efficacy relationship using modeling in Alzheimer’s disease. Alzheimers Dement (N Y) 9:e12404. 10.1002/trc2.12404 37388759 10.1002/trc2.12404 PMC10301702

[41] Güttler BH-O, Cynis H, Seifert F, Ludwig H-H, Porzel A, Schilling S (2013) A quantitative analysis of spontaneous isoaspartate formation from N-terminal asparaginyl and aspartyl residues. Amino Acids 44:1205–1214. 10.1007/s00726-012-1454-0 23344882 10.1007/s00726-012-1454-0

[42] Hamilton RL (2000) Lewy bodies in Alzheimer’s disease: a neuropathological review of 145 cases using alpha-synuclein immunohistochemistry. Brain Pathol 10:378–384. 10.1111/j.1750-3639.2000.tb00269.x 10885656 10.1111/j.1750-3639.2000.tb00269.x PMC8098522

[43] Hardy J, Schott JM (2024) Identifying genetic risk for amyloid-related imaging abnormalities. Neurology 102:e208096. 10.1212/WNL.0000000000208096 38165303 10.1212/WNL.0000000000208096

[44] Hartlage-Rübsamen M, Morawski M, Waniek A, Jäger C, Zeitschel U, Koch B et al (2011) Glutaminyl cyclase contributes to the formation of focal and diffuse pyroglutamate (pGlu)-Aβ deposits in hippocampus via distinct cellular mechanisms. Acta Neuropathol 121:705–719. 10.1007/s00401-011-0806-2 21301857 10.1007/s00401-011-0806-2 PMC3098988

[45] Hartlage-Rübsamen M, Bluhm A, Piechotta A, Linnert M, Rahfeld J-U, Demuth H-U et al (2018) Immunohistochemical evidence from APP-transgenic mice for glutaminyl cyclase as drug target to diminish pE-Abeta formation. Molecules 23:924. 10.3390/molecules23040924 29673150 10.3390/molecules23040924 PMC6017857

[46] Hoffmann T, Meyer A, Heiser U, Kurat S, Böhme L, Kleinschmidt M et al (2017) Glutaminyl cyclase inhibitor PQ912 improves cognition in mouse models of Alzheimer’s disease-studies on relation to effective target occupancy. J Pharmacol Exp Ther 362:119–130. 10.1124/jpet.117.240614 28446518 10.1124/jpet.117.240614

[47] Inoue K, Hosaka D, Mochizuki N, Akatsu H, Tsutsumiuchi K, Hashizume Y et al (2014) Simultaneous determination of post-translational racemization and isomerization of N-terminal amyloid-β in Alzheimer’s brain tissues by covalent chiral derivatized ultraperformance liquid chromatography tandem mass spectrometry. Anal Chem 86:797–804. 10.1021/ac403315h 24283798 10.1021/ac403315h

[48] Jan A, Gokce O, Luthi-Carter R, Lashuel HA (2008) The ratio of monomeric to aggregated forms of Aβ40 and Aβ42 is an important determinant of amyloid-β aggregation, fibrillogenesis, and toxicity. J Biol Chem 283:28176–28189. 10.1074/jbc.M803159200 18694930 10.1074/jbc.M803159200 PMC2661389

[49] Jarrett JT, Berger EP, Lansbury PT (1993) The carboxy terminus of the beta amyloid protein is critical for the seeding of amyloid formation: implications for the pathogenesis of Alzheimer’s disease. Biochemistry 32:4693–4697. 10.1021/bi00069a001 8490014 10.1021/bi00069a001

[50] Jucker M, Walker LC (2023) Alzheimer’s disease: from immunotherapy to immunoprevention. Cell 186:4260–4270. 10.1016/j.cell.2023.08.021 37729908 10.1016/j.cell.2023.08.021 PMC10578497

[51] Kang C (2024) Donanemab: first approval. Drugs. 10.1007/s40265-024-02087-4 39404937 10.1007/s40265-024-02103-7

[52] Kleinschmidt M, Schilling S, Rahfeld J-U et al. (2012) Diagnostic Antibody Assay(WO/2012/123562). https://patents.google.com/patent/DK2686346T3/da?oq=DK2686346T3. Accessed 19 Jun 2024

[53] Köppen J, Schulze A, Machner L, Wermann M, Eichentopf R, Guthardt M et al (2020) Amyloid-beta peptides trigger aggregation of alpha-synuclein in vitro. Molecules 25:580. 10.3390/molecules25030580 32013170 10.3390/molecules25030580 PMC7037551

[54] Kozin SA, Cheglakov IB, Ovsepyan AA, Telegin GB, Tsvetkov PO, Lisitsa AV et al (2013) Peripherally applied synthetic peptide isoAsp7-Aβ(1–42) triggers cerebral β-amyloidosis. Neurotox Res 24:370–376. 10.1007/s12640-013-9399-y 23670398 10.1007/s12640-013-9399-y

[55] Kumar S, Rezaei-Ghaleh N, Terwel D, Thal DR, Richard M, Hoch M et al (2011) Extracellular phosphorylation of the amyloid β-peptide promotes formation of toxic aggregates during the pathogenesis of Alzheimer’s disease. EMBO J 30:2255–2265. 10.1038/emboj.2011.138 21527912 10.1038/emboj.2011.138 PMC3117653

[56] Kumar S, Wirths O, Theil S, Gerth J, Bayer TA, Walter J (2013) Early intraneuronal accumulation and increased aggregation of phosphorylated Abeta in a mouse model of Alzheimer’s disease. Acta Neuropathol 125:699–709. 10.1007/s00401-013-1107-8 23525537 10.1007/s00401-013-1107-8

[57] Kumar S, Wirths O, Stüber K, Wunderlich P, Koch P, Theil S et al (2016) Phosphorylation of the amyloid β-peptide at Ser26 stabilizes oligomeric assembly and increases neurotoxicity. Acta Neuropathol 131:525–537. 10.1007/s00401-016-1546-0 26898910 10.1007/s00401-016-1546-0 PMC4789232

[58] Kumar S, Frost JL, Cotman CW, Head E, Palmour R, Lemere CA et al (2018) Deposition of phosphorylated amyloid-β in brains of aged nonhuman primates and canines. Brain Pathol 28:427–430. 10.1111/bpa.12573 29740941 10.1111/bpa.12573 PMC8028592

[59] Kumar S, Kapadia A, Theil S, Joshi P, Riffel F, Heneka MT et al (2020) Novel phosphorylation-state specific antibodies reveal differential deposition of Ser26 phosphorylated Aβ species in a mouse model of Alzheimer’s disease. Front Mol Neurosci 13:619639. 10.3389/fnmol.2020.619639 33519377 10.3389/fnmol.2020.619639 PMC7844098

[60] Kumar S, Lemere CA, Walter J (2020) Phosphorylated Aβ peptides in human Down syndrome brain and different Alzheimer’s-like mouse models. Acta Neuropathol Commun 8:118. 10.1186/s40478-020-00959-w 32727580 10.1186/s40478-020-00959-w PMC7388542

[61] Kummer MP, Hermes M, Delekarte A, Hammerschmidt T, Kumar S, Terwel D et al (2011) Nitration of tyrosine 10 critically enhances amyloid β aggregation and plaque formation. Neuron 71:833–844. 10.1016/j.neuron.2011.07.001 21903077 10.1016/j.neuron.2011.07.001

[62] Kummer MP, Heneka MT (2014) Truncated and modified amyloid-beta species. Alzheimers Res Ther 6:28. 10.1186/alzrt258 25031638 10.1186/alzrt258 PMC4055046

[63] Kuo YM, Emmerling MR, Woods AS, Cotter RJ, Roher AE (1997) Isolation, chemical characterization, and quantitation of Abeta 3-pyroglutamyl peptide from neuritic plaques and vascular amyloid deposits. Biochem Biophys Res Commun 237:188–191. 10.1006/bbrc.1997.7083 9266855 10.1006/bbrc.1997.7083

[64] Landles C, Sathasivam K, Weiss A, Woodman B, Moffitt H, Finkbeiner S et al (2010) Proteolysis of mutant huntingtin produces an exon 1 fragment that accumulates as an aggregated protein in neuronal nuclei in Huntington disease. J Biol Chem 285:8808–8823. 10.1074/jbc.M109.075028 20086007 10.1074/jbc.M109.075028 PMC2838303

[65] Mandler M, Walker L, Santic R, Hanson P, Upadhaya AR, Colloby SJ et al (2014) Pyroglutamylated amyloid-β is associated with hyperphosphorylated tau and severity of Alzheimer’s disease. Acta Neuropathol 128:67–79. 10.1007/s00401-014-1296-9 24861310 10.1007/s00401-014-1296-9

[66] Masters CL, Simms G, Weinman NA, Multhaup G, McDonald BL, Beyreuther K (1985) Amyloid plaque core protein in Alzheimer disease and Down syndrome. Proc Natl Acad Sci U S A 82:4245–4249. 10.1073/pnas.82.12.4245 3159021 10.1073/pnas.82.12.4245 PMC397973

[67] McKeith IG, Boeve BF, Dickson DW, Halliday G, Taylor J-P, Weintraub D et al (2017) Diagnosis and management of dementia with Lewy bodies: fourth consensus report of the DLB consortium. Neurology 89:88–100. 10.1212/WNL.0000000000004058 28592453 10.1212/WNL.0000000000004058 PMC5496518

[68] Miller DL, Papayannopoulos IA, Styles J, Bobin SA, Lin YY, Biemann K et al (1993) Peptide compositions of the cerebrovascular and senile plaque core amyloid deposits of Alzheimer’s disease. Arch Biochem Biophys 301:41–52. 10.1006/abbi.1993.1112 8442665 10.1006/abbi.1993.1112

[69] Mitkevich VA, Petrushanko IY, Yegorov YE, Simonenko OV, Vishnyakova KS, Kulikova AA et al (2013) Isomerization of Asp7 leads to increased toxic effect of amyloid-β42 on human neuronal cells. Cell Death Dis 4:e939. 10.1038/cddis.2013.492 24287700 10.1038/cddis.2013.492 PMC3847340

[70] Montalbano M, Majmundar L, Sengupta U, Fung L, Kayed R (2022) Pathological tau signatures and nuclear alterations in neurons, astrocytes and microglia in Alzheimer’s disease, progressive supranuclear palsy, and dementia with Lewy bodies. Brain Pathol 33:e13112. 10.1111/bpa.13112 36054524 10.1111/bpa.13112 PMC9836371

[71] Morawski M, Hartlage-Rübsamen M, Jäger C, Waniek A, Schilling S, Schwab C et al (2010) Distinct glutaminyl cyclase expression in Edinger-Westphal nucleus, locus coeruleus and nucleus basalis Meynert contributes to pGlu-Abeta pathology in Alzheimer’s disease. Acta Neuropathol 120:195–207. 10.1007/s00401-010-0685-y 20383514 10.1007/s00401-010-0685-y PMC2892616

[72] Morawski M, Schilling S, Kreuzberger M, Waniek A, Jäger C, Koch B et al (2014) Glutaminyl cyclase in human cortex: correlation with (pGlu)-amyloid-β load and cognitive decline in Alzheimer’s disease. J Alzheimers Dis 39:385–400. 10.3233/JAD-131535 24164736 10.3233/JAD-131535

[73] Mori C, Spooner ET, Wisniewsk KE, Wisniewski TM, Yamaguch H, Saido TC et al (2002) Intraneuronal Abeta42 accumulation in Down syndrome brain. Amyloid 9:88–102 12440481

[74] Moro ML, Collins MJ, Cappellini E (2010) Alzheimer’s disease and amyloid beta-peptide deposition in the brain: a matter of “aging”? Biochem Soc Trans 38:539–544. 10.1042/BST0380539 20298218 10.1042/BST0380539

[75] Moro ML, Phillips AS, Gaimster K, Paul C, Mudher A, Nicoll JAR et al (2018) Pyroglutamate and isoaspartate modified amyloid-beta in ageing and Alzheimer’s disease. Acta Neuropathol Commun 6:3. 10.1186/s40478-017-0505-x 29298722 10.1186/s40478-017-0505-x PMC5753481

[76] Mukherjee S, Perez KA, Lago LC, Klatt S, McLean CA, Birchall IE et al (2021) Quantification of N-terminal amyloid-β isoforms reveals isomers are the most abundant form of the amyloid-β peptide in sporadic Alzheimer’s disease. Brain Commun 3:fcab028. 10.1093/braincomms/fcab028 33928245 10.1093/braincomms/fcab028 PMC8062259

[77] Nussbaum JM, Schilling S, Cynis H, Silva A, Swanson E, Wangsanut T et al (2012) Prion-like behaviour and tau-dependent cytotoxicity of pyroglutamylated amyloid-β. Nature 485:651–655. 10.1038/nature11060 22660329 10.1038/nature11060 PMC3367389

[78] Orpiszewski J, Schormann N, Kluve-Beckerman B, Liepnieks JJ, Benson MD (2000) Protein aging hypothesis of Alzheimer disease. FASEB J 14:1255–1263. 10.1096/fasebj.14.9.1255 10834947 10.1096/fasebj.14.9.1255

[79] Perez-Garmendia R, Gevorkian G (2013) Pyroglutamate-modified amyloid beta peptides: emerging targets for Alzheimer’s disease immunotherapy. Curr Neuropharmacol 11:491–498. 10.2174/1570159X11311050004 24403873 10.2174/1570159X11311050004 PMC3763757

[80] Piechotta A, Parthier C, Kleinschmidt M, Gnoth K, Pillot T, Lues I et al (2017) Structural and functional analyses of pyroglutamate-amyloid-β-specific antibodies as a basis for Alzheimer immunotherapy. J Biol Chem 292:12713–12724. 10.1074/jbc.M117.777839 28623233 10.1074/jbc.M117.777839 PMC5535044

[81] Pike CJ, Burdick D, Walencewicz AJ, Glabe CG, Cotman CW (1993) Neurodegeneration induced by beta-amyloid peptides in vitro: the role of peptide assembly state. J Neurosci 13:1676–1687. 10.1523/JNEUROSCI.13-04-01676.1993 8463843 10.1523/JNEUROSCI.13-04-01676.1993 PMC6576726

[82] Plant LD, Boyle JP, Smith IF, Peers C, Pearson HA (2003) The production of amyloid beta peptide is a critical requirement for the viability of central neurons. J Neurosci 23:5531–5535. 10.1523/JNEUROSCI.23-13-05531.2003 12843253 10.1523/JNEUROSCI.23-13-05531.2003 PMC6741264

[83] Portelius E, Bogdanovic N, Gustavsson MK, Volkmann I, Brinkmalm G, Zetterberg H et al (2010) Mass spectrometric characterization of brain amyloid beta isoform signatures in familial and sporadic Alzheimer’s disease. Acta Neuropathol 120:185–193. 10.1007/s00401-010-0690-1 20419305 10.1007/s00401-010-0690-1 PMC3568930

[84] Puzzo D, Privitera L, Fa’ M, Staniszewski A, Hashimoto G, Aziz F et al (2011) Endogenous amyloid-β is necessary for hippocampal synaptic plasticity and memory. Ann Neurol 69:819–830. 10.1002/ana.22313 21472769 10.1002/ana.22313 PMC4071456

[85] Racine AM, Koscik RL, Nicholas CR, Clark LR, Okonkwo OC, Oh JM et al (2016) Cerebrospinal fluid ratios with Aβ42 predict preclinical brain β-amyloid accumulation. Alzheimers Dement (Amst) 2:27–38. 10.1016/j.dadm.2015.11.006 26955655 10.1016/j.dadm.2015.11.006 PMC4778249

[86] Recchia A, Debetto P, Negro A, Guidolin D, Skaper SD, Giusti P (2004) α-synuclein and Parkinson’s disease. FASEB J 18:617–626. 10.1096/fj.03-0338rev 15054084 10.1096/fj.03-0338rev

[87] Revesz T, Holton JL, Lashley T, Plant G, Frangione B, Rostagno A et al (2009) Genetics and molecular pathogenesis of sporadic and hereditary cerebral amyloid angiopathies. Acta Neuropathol 118:115–130. 10.1007/s00401-009-0501-8 19225789 10.1007/s00401-009-0501-8 PMC2844092

[88] Rezaei-Ghaleh N, Amininasab M, Giller K, Kumar S, Stündl A, Schneider A et al (2014) Turn plasticity distinguishes different modes of amyloid-β aggregation. J Am Chem Soc 136:4913–4919. 10.1021/ja411707y 24617810 10.1021/ja411707y

[89] Rijal UA, Kosterin I, Kumar S, Von ACA, Yamaguchi H, Fändrich M et al (2014) Biochemical stages of amyloid-β peptide aggregation and accumulation in the human brain and their association with symptomatic and pathologically preclinical Alzheimer’s disease. Brain 137:887–903. 10.1093/brain/awt362 24519982 10.1093/brain/awt362

[90] Roher AE, Lowenson JD, Clarke S, Wolkow C, Wang R, Cotter RJ et al (1993) Structural alterations in the peptide backbone of beta-amyloid core protein may account for its deposition and stability in Alzheimer’s disease. J Biol Chem 268:3072–3083 8428986

[91] Roher AE, Kokjohn TA, Clarke SG, Sierks MR, Maarouf CL, Serrano GE et al (2017) APP/Aβ structural diversity and Alzheimer’s disease pathogenesis. Neurochem Int 110:1–13. 10.1016/j.neuint.2017.08.007 28811267 10.1016/j.neuint.2017.08.007 PMC5688956

[92] Román GC, Tatemichi TK, Erkinjuntti T, Cummings JL, Masdeu JC, Garcia JH et al (1993) Vascular dementia: diagnostic criteria for research studies. Report of the NINDS-AIREN international WORKSHOP. Neurology 43:250–260. 10.1212/wnl.43.2.250 8094895 10.1212/wnl.43.2.250

[93] Rosen RF, Tomidokoro Y, Farberg AS, Dooyema J, Ciliax B, Preuss TM et al (2016) Comparative pathobiology of β-amyloid and the unique susceptibility of humans to Alzheimer’s disease. Neurobiol Aging 44:185–196. 10.1016/j.neurobiolaging.2016.04.019 27318146 10.1016/j.neurobiolaging.2016.04.019 PMC4913040

[94] Rostagno A, Cabrera E, Lashley T, Ghiso J (2022) N-terminally truncated Aβ4-x proteoforms and their relevance for Alzheimer’s pathophysiology. Transl Neurodegener 11:30. 10.1186/s40035-022-00303-3 35641972 10.1186/s40035-022-00303-3 PMC9158284

[95] Russo C, Violani E, Salis S, Venezia V, Dolcini V, Damonte G et al (2002) Pyroglutamate-modified amyloid beta-peptides - AbetaN3(pE) - strongly affect cultured neuron and astrocyte survival. J Neurochem 82:1480–1489. 10.1046/j.1471-4159.2002.01107.x 12354296 10.1046/j.1471-4159.2002.01107.x

[96] Saido TC (1998) Alzheimer’s disease as proteolytic disorders: anabolism and catabolism of beta-amyloid. Neurobiol Aging 19:S69–S75. 10.1016/s0197-4580(98)00033-5 9562472 10.1016/s0197-4580(98)00033-5

[97] Saido TC, Iwatsubo T, Mann DM, Shimada H, Ihara Y, Kawashima S (1995) Dominant and differential deposition of distinct beta-amyloid peptide species, Abeta N3(pE), in senile plaques. Neuron 14:457–466. 10.1016/0896-6273(95)90301-1 7857653 10.1016/0896-6273(95)90301-1

[98] Scheidt HA, Adler J, Zeitschel U, Höfling C, Korn A, Krueger M et al (2017) Pyroglutamate-modified amyloid β (11–40) fibrils are more toxic than wildtype fibrils but structurally very similar. Chemistry 23:15834–15838. 10.1002/chem.201703909 28857302 10.1002/chem.201703909

[99] Scheidt HA, Das A, Korn A, Krueger M, Maiti S, Huster D (2020) Structural characteristics of oligomers formed by pyroglutamate-modified amyloid β peptides studied by solid-state NMR. Phys Chem Chem Phys 22:16887–16895. 10.1039/d0cp02307h 32666970 10.1039/d0cp02307h

[100] Scheltens P, Hallikainen M, Grimmer T, Duning T, Gouw AA, Teunissen CE et al (2018) Safety, tolerability and efficacy of the glutaminyl cyclase inhibitor PQ912 in Alzheimer’s disease: results of a randomized, double-blind, placebo-controlled phase 2a study. Alzheimers Res Ther 10:107. 10.1186/s13195-018-0431-6 30309389 10.1186/s13195-018-0431-6 PMC6182869

[101] Schilling S, Lauber T, Schaupp M, Manhart S, Scheel E, Böhm G et al (2006) On the seeding and oligomerization of pGlu-amyloid peptides (in vitro). Biochemistry 45:12393–12399. 10.1021/bi0612667 17029395 10.1021/bi0612667

[102] Schilling G, Klevytska A, Tebbenkamp ATN, Juenemann K, Cooper J, Gonzales V et al (2007) Characterization of huntingtin pathologic fragments in human Huntington disease, transgenic mice, and cell models. J Neuropathol Exp Neurol 66:313–320. 10.1097/nen.0b013e318040b2c8 17413322 10.1097/nen.0b013e318040b2c8

[103] Schilling S, Zeitschel U, Hoffmann T, Heiser U, Francke M, Kehlen A et al (2008) Glutaminyl cyclase inhibition attenuates pyroglutamate Aβ and Alzheimer’s disease–like pathology. Nat Med 14:1106–1111. 10.1038/nm.1872 18836460 10.1038/nm.1872

[104] Schlenzig D, Manhart S, Cinar Y, Kleinschmidt M, Hause G, Willbold D et al (2009) Pyroglutamate formation influences solubility and amyloidogenicity of amyloid peptides. Biochemistry 48:7072–7078. 10.1021/bi900818a 19518051 10.1021/bi900818a

[105] Schober P, Boer C, Schwarte LA (2018) Correlation coefficients: appropriate use and interpretation. Anesth Analg 126:1763–1768. 10.1213/ANE.0000000000002864 29481436 10.1213/ANE.0000000000002864

[106] Schumacher J, Gunter JL, Przybelski SA, Jones DT, Graff-Radford J, Savica R et al (2021) Dementia with Lewy bodies: association of Alzheimer pathology with functional connectivity networks. Brain 144:3212–3225. 10.1093/brain/awab218 34114602 10.1093/brain/awab218 PMC8634124

[107] Shin Y, Cho HS, Fukumoto H, Shimizu T, Shirasawa T, Greenberg SM et al (2003) Aβ species, including IsoAsp23 Abeta, in Iowa-type familial cerebral amyloid angiopathy. Acta Neuropathol 105:252–258. 10.1007/s00401-002-0639-0 12557012 10.1007/s00401-002-0639-0

[108] Sims JR, Zimmer JA, Evans CD, Lu M, Ardayfio P, Sparks J et al (2023) Donanemab in early symptomatic Alzheimer disease: the TRAILBLAZER-ALZ 2 randomized clinical trial. JAMA 330:512–527. 10.1001/jama.2023.13239 37459141 10.1001/jama.2023.13239 PMC10352931

[109] Soto-Rojas LO, Campa-Córdoba BB, Harrington CR, Salas-Casas A, Hernandes-Alejandro M, Villanueva-Fierro I et al (2021) Insoluble vascular amyloid deposits trigger disruption of the neurovascular unit in Alzheimer’s disease brains. Int J Mol Sci 22:3654. 10.3390/ijms22073654 33915754 10.3390/ijms22073654 PMC8036769

[110] Spillantini MG, Schmidt ML, Lee VM, Trojanowski JQ, Jakes R, Goedert M (1997) α-synuclein in Lewy bodies. Nature 388:839–840. 10.1038/42166 9278044 10.1038/42166

[111] Sullivan CP, Berg EA, Elliott-Bryant R, Fishman JB, McKee AC, Morin PJ et al (2011) Pyroglutamate-Aβ 3 and 11 colocalize in amyloid plaques in Alzheimer’s disease cerebral cortex with pyroglutamate-Aβ 11 forming the central core. Neurosci Lett 505:109–112. 10.1016/j.neulet.2011.09.071 22001577 10.1016/j.neulet.2011.09.071 PMC3253715

[112] Thal WJ, Saido TC, Fändrich M (2015) Neuropathology and biochemistry of Aβ and its aggregates in Alzheimer’s disease. Acta Neuropathol 129:167–182. 10.1007/s00401-014-1375-y 25534025 10.1007/s00401-014-1375-y

[113] Thal DR, Rüb U, Orantes M, Braak H (2002) Phases of Aβ-deposition in the human brain and its relevance for the development of AD. Neurology 58:1791–1800. 10.1212/wnl.58.12.1791 12084879 10.1212/wnl.58.12.1791

[114] Tomidokoro Y, Lashley T, Rostagno A, Neubert TA, Bojsen-Møller M, Braendgaard H et al (2005) Familial Danish dementia: co-existence of Danish and Alzheimer amyloid subunits (ADan and Aβ) in the absence of compact plaques. J Biol Chem 280:36883–36894. 10.1074/jbc.M504038200 16091362 10.1074/jbc.M504038200

[115] Tomidokoro Y, Rostagno A, Neubert TA, Lu Y, Rebeck GW, Frangione B et al (2010) Iowa variant of familial Alzheimer’s disease: accumulation of posttranslationally modified AβD23N in parenchymal and cerebrovascular amyloid deposits. Am J Pathol 176:1841–1854. 10.2353/ajpath.2010.090636 20228223 10.2353/ajpath.2010.090636 PMC2843474

[116] Tu PH, Galvin JE, Baba M, Giasson B, Tomita T, Leight S et al (1998) Glial cytoplasmic inclusions in white matter oligodendrocytes of multiple system atrophy brains contain insoluble α-synuclein. Ann Neurol 44:415–422. 10.1002/ana.410440324 9749615 10.1002/ana.410440324

[117] van der Gaag BL, Deshayes NAC, Breve JJP, Bol JGJM, Jonker AJ, Hoozemans JJM et al (2024) Distinct tau and alpha-synuclein molecular signatures in Alzheimer’s disease with and without Lewy bodies and Parkinson’s disease with dementia. Acta Neuropathol 147:14. 10.1007/s00401-023-02657-y 38198008 10.1007/s00401-023-02657-y PMC10781859

[118] van Wetering J, Geut H, Bol JJ, Galis Y, Timmermans E, Twisk JWR et al (2024) Neuroinflammation is associated with Alzheimer’s disease co-pathology in dementia with Lewy bodies. Acta Neuropathol Commun 12:73. 10.1186/s40478-024-01786-z 38715119 10.1186/s40478-024-01786-z PMC11075309

[119] Vijverberg EGB, Axelsen TM, Bihlet AR, Henriksen K, Weber F, Fuchs K et al (2021) Rationale and study design of a randomized, placebo-controlled, double-blind phase 2b trial to evaluate efficacy, safety, and tolerability of an oral glutaminyl cyclase inhibitor varoglutamstat (PQ912) in study participants with MCI and mild AD—VIVIAD. Alzheimers Res Ther 13:142. 10.1186/s13195-021-00882-9 34425883 10.1186/s13195-021-00882-9 PMC8381483

[120] Walter S, Jumpertz T, Hüttenrauch M, Ogorek I, Gerber H, Storck SE et al (2019) The metalloprotease ADAMTS4 generates N-truncated Aβ4-x species and marks oligodendrocytes as a source of amyloidogenic peptides in Alzheimer’s disease. Acta Neuropathol 137:239–257. 10.1007/s00401-018-1929-5 30426203 10.1007/s00401-018-1929-5

[121] Weggen S, Beher D (2012) Molecular consequences of amyloid precursor protein and presenilin mutations causing autosomal-dominant Alzheimer’s disease. Alzheimers Res Ther 4:9. 10.1186/alzrt107 22494386 10.1186/alzrt107 PMC3334542

[122] Wirths O, Bethge T, Marcello A, Harmeier A, Jawhar S, Lucassen PJ et al (2010) Pyroglutamate Abeta pathology in APP/PS1KI mice, sporadic and familial Alzheimer’s disease cases. J Neural Transm (Vienna) 117:85–96. 10.1007/s00702-009-0314-x 19823761 10.1007/s00702-009-0314-x PMC2789212

[123] Wirths O, Walter S, Kraus I, Klafki HW, Stazi M, Oberstein TJ et al (2017) N-truncated Aβ4-x peptides in sporadic Alzheimer’s disease cases and transgenic Alzheimer mouse models. Alzheimers Res Ther 9:80. 10.1186/s13195-017-0309-z 28978359 10.1186/s13195-017-0309-z PMC5628465

[124] Yang Y, Arseni D, Zhang W, Huang M, Lövestam S, Schweighauser M et al (2022) Cryo-EM structures of amyloid-β 42 filaments from human brains. Science 375:167–172. 10.1126/science.abm7285 35025654 10.1126/science.abm7285 PMC7612234

[125] Zampar S, Klafki HW, Sritharen K, Bayer TA, Wiltfang J, Rostagno A et al (2020) N-terminal heterogeneity of parenchymal and vascular amyloid-β deposits in Alzheimer’s disease. Neuropathol Appl Neurobiol 46:673–685. 10.1111/nan.12637 32497293 10.1111/nan.12637 PMC8082844

[126] Zhang-Nunes SX, Maat-Schieman MLC, van Duinen SG, Roos RAC, Frosch MP, Greenberg SM (2006) The cerebral β-amyloid angiopathies: hereditary and sporadic. Brain Pathol 16:30–39. 10.1111/j.1750-3639.2006.tb00559.x 16612980 10.1111/j.1750-3639.2006.tb00559.x PMC8095991

