# Supplementary material for: Identification of isoAsp7-Aβ as a major Aβ variant in Alzheimer’s disease, dementia with Lewy bodies and vascular dementia
Source: Acta Neuropathol. 2024 Dec 3;148(1):78. doi: 10.1007/s00401-024-02824-9 (PMC11615120; doi:10.1007/s00401-024-02824-9)
Supplement: Supplementary file 1 — Supplementary file1 (DOCX 32273 KB) [file 401_2024_2824_MOESM1_ESM.docx]

**Supplementary Information to**

**Identification of isoAsp7-Aβ as a major Aβ variant in Alzheimer’s disease, dementia with Lewy bodies and vascular dementia**

Sarah Schrempel^1,^*, Anna Katharina Kottwitz^2,3,^*, Anke Piechotta^2^, Kathrin Gnoth^2,3^,

Luca Büschgens^4^, Maike Hartlage-Rübsamen^1^, Markus Morawski^1^, Mathias Schenk^2^, Martin Kleinschmidt^2^, Geidy E. Serrano^5^, Thomas G. Beach^5^, Agueda Rostagno^6^, Jorge Ghiso^6^, Michael T. Heneka^7^, Jochen Walter^8^, Oliver Wirths^4^,

Stephan Schilling^2,3^, Steffen Roßner^1§^

*1 Paul Flechsig Institute – Centre of Neuropathology and Brain Research, University of Leipzig, 04103 Leipzig, Germany*

*2 Fraunhofer Institute for Cell Therapy and Immunology, Department of Molecular Drug Design and Target Validation, 06120 Halle (Saale), Germany*

*3 Anhalt University of Applied Sciences, Center for Natural Products-based Therapeutics, 06366 Köthen, Germany*

*4 Department of Psychiatry and Psychotherapy, University Medical Center Göttingen, Georg-August-University, 37075 Göttingen, Germany*

*5 Civin Laboratory for Neuropathology, Brain and Body Donation Program Banner Sun Health Research Institute, 10515 W Santa Fe Drive, Sun City, AZ 85351, USA*

*6 Department of Pathology, New York University School of Medicine, New York, NY, USA*

*7 Luxembourg Centre for Systems Biomedicine, University of Luxembourg, Belval, Luxembourg*

*8 Center of Neurology, Molecular Cell Biology, University Hospital Bonn, 53127 Bonn, Germany*

* These authors contributed equally to the manuscript.

**Submitted to:** Acta Neuropathologica

**Generation of antibodies against 3NTyr10-Aβ and isoAsp27-Aβ**

The 3NTyr10-Aβ-specific antibody 4C3 was generated by immunization of female BALB/c mice with the chemically synthesized peptide 3NTyr10-Aβ(6-14) conjugated to keyhole limpet hemocyanin (BioGenes GmbH, Berlin, Germany). Hybridoma cell clones were produced by fusion of spleen cells of the immunized mice with the myeloma cell line SP2/0. Clones were selected using immunoassay measurements. Recombinant expression of 4C3 in ExpiCHO-S cells (Gibco, Thermo Fisher Scientific, Waltham, MA, USA) using vector pTRIOZ-hIgG1 (Invitrogen, Thermo Fisher Scientific), that was modified to pTRIOZ-mIgG2a, was used for antibody production. All antibodies generated by Fraunhofer IZI were purified from hybridoma, Freestyle 293-F or ExpiCHO expression medium supernatant by Protein G affinity chromatography (Cytiva, Marlborough, MA, USA). Bound antibodies were eluted using 100 mM Glycine-HCl, pH 2.7, or using 2 M KSCN, 40 mM Na_2_HPO_4_, pH 7.0 and dialyzed twice against PBS (138 mM NaCl, 8 mM Na_2_HPO_4_, 1.5 mM KH_2_PO_4_, 3 mM KCl, pH 7.1) at 4°C overnight.

The antibody F2 against isoAsp27-Aβ was recombinantly expressed in Freestyle 293-F cells (Thermo Fisher Scientific) by using the bicistronic vector pVITRO-neo-mcs2. For this purpose, the sequence of F2 was derived from murine hybridoma cells generated by UNICUS Karlsburg OHG (Karlsburg, Germany) using the immunization peptide isoAsp27-Aβ(23-33).

**Characterization of the novel monoclonal 3NTyr10-Aβ antibody 4C3**

With the aim of testing the binding capacity of the novel 3NTyr10-Aβ antibody 4C3, an immunoassay based on different C-terminally truncated 3NTyr10-Aβ species as well as wild type Aβ was used. The concentration of 3NTyr-containing analogs was determined by UV-Vis at 381 nm using a molar extinction coefficient of 2200 M^-1^ cm^-1^ [1]. First, synthetic peptides were serially diluted in PBS and immobilized on polystyrene 96-well microtiter plates (Nunc Maxisorp, flat-bottom) at 4°C overnight. After blocking with TBS ELISA Blocker (Thermo Fisher Scientific) at 4°C for 2 h, washing was performed three times with TBS-T. Next, synthetic peptides were incubated with primary antibodies in appropriate dilutions (2 µg/mL 4C3; 0.4 µg/mL 3D6 and 0.4 µg/mL 39B6) in TBS ELISA Blocker + Tween 20 at 4°C for 2 h. The antibody 39B6, which recognizes nitrated tyrosine (3NTyr) irrespective of the surrounding amino acid sequence, was purchased from Santa Cruz Biotechnology (sc-32757; Santa Cruz Biotechnology, Inc., Dallas, TX, USA). Plates were washed six times with TBS-T. For detection of bound antibodies, cross-adsorbed goat anti mouse IgG (H+L) HRP (Invitrogen, Thermo Fisher Scientific) was diluted to a final concentration of 0.2 µg/mL in ELISA Blocker + Tween 20 and incubated with the samples at 4°C for 1 h. After six washing steps with TBS-T, a color reaction with commercially available HRP substrate TMB (SureBlue Reserve TMB Microwell Peroxidase Substrate (1-component); KPL, LGC Clinical Diagnostics, Milford, MA, USA) was performed as described above.

The antibody 4C3 against 3NTyr10-Aβ recognized the 3NTyr10-Aβ(1-16) but not the Aβ(1-18) fragment (**Suppl. Fig. 1,** left). As a control, the sequence-independent 3NTyr antibody 39B6 was used and also shown to only bind 3NTyr10-Aβ (1-16) fragments (**Suppl. Fig. 1,** middle). In contrast, the antibody 3D6, raised against amino acid 1 to 5 of Aβ, recognized both Aβ(1-18) and 3NTyr10-Aβ (1-16) fragments (**Suppl. Fig. 1,** right).


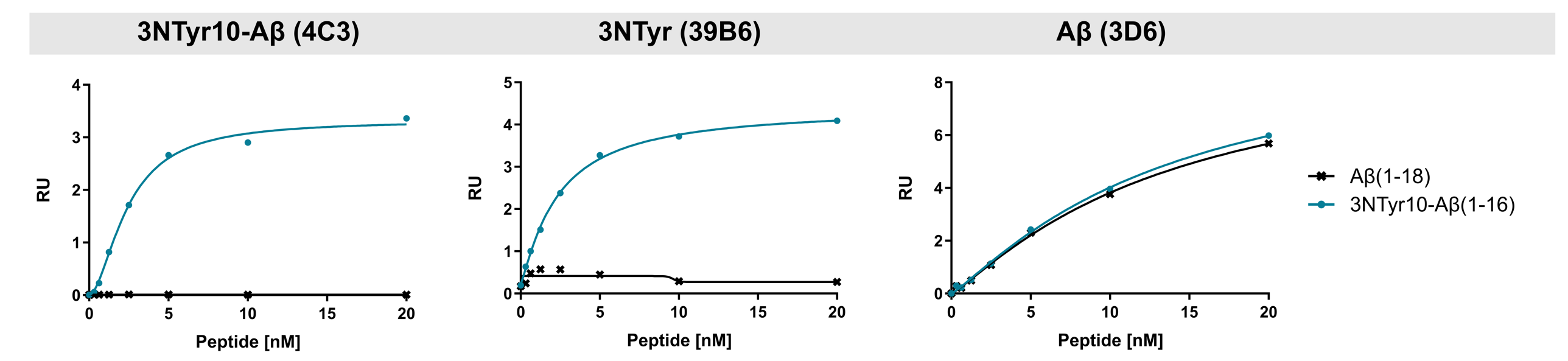


**Suppl. Fig. 1:** Immunoassay analyses with varying concentrations of the peptides Aβ(1-18) (black lines) and 3NTyr10-Aβ(1-16) (blue lines) using the novel 3NTyr10-Aβ antibody 4C3 (left), the general 3NTyr antibody 39B6 (middle) and the Aβ antibody 3D6 (right). Note the specificity of the 4C3 antibody against 3NTyr10-Aβ.

**Characterization of the monoclonal isoAsp27-Aβ antibody F2**

Initial testing of the specificity of the novel isoAsp27-Aβ antibody F2 was performed by immunoassay in comparable manner to the 3NTyr10-Aβ immunoassay. Therefore, Aβ(19-34) species containing an isoAsp at position 27 were used. During peptide synthesis, biotin was added to the C-terminus via polyethylene glycol (Pbio), which enables the immobilization of the peptides using streptavidin. As a first step, 0.4 µg/well streptavidin (Carl Roth GmbH + Co. KG, Karlsruhe, Germany) in PBS were immobilized on polystyrene 96-well microtiter plates at 4°C overnight. After blocking at 4°C for 2 h, the peptides were serially diluted and incubated at 4°C for 2 h. F2 (2 µg/ml) was used as primary antibody. For detection of bound antibodies, cross-adsorbed Goat anti Mouse IgG (H+L) HRP (Invitrogen, Thermo Fisher Scientific) was diluted to a final concentration of 0.2 µg/mL and incubated with the samples at 4°C for 1 h. The color reaction with commercially available HRP substrate TMB was performed as described above.

The antibody F2 against isoAsp27-Aβ recognized the isoAsp27-Aβ(19-34) but not the Aβ(19-34) fragment (**Suppl. Fig. 2**).


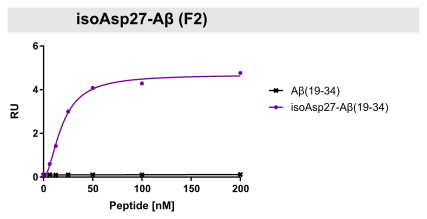


**Suppl. Fig. 2:** Immunoassay analyses with varying concentrations of the peptides Aβ(19-34) (black line) and isoAsp27-Aβ(19-34) (purple line) using the novel isoAsp27-Aβ antibody F2. Note the specificity of the F2 antibody against isoAsp27-Aβ.

In **Suppl. Table 1**, the sequence and origin of all peptides used for immunization of mice and as standards for immunoassay analyses are listed.

**Suppl. Table 1:** Synthetic standard peptides for immunoassay analyses and immunization peptides for antibody generation

| **Peptide** | **Sequence** | **Origin** |
| --- | --- | --- |
| Aβ(1-18) | H-DAEFRHDSGYEVHHQKLV-NH2 | Fraunhofer IZI (Halle, Germany) |
| Aβ(1-40) | H-DAEFRHDSGYEVHHQKLVFFAEDVGSNKGAIIGLMVGGVV-OH | Fraunhofer IZI (Halle, Germany) |
| Aβ(4-40) | H-FRHDSGYEVHHQKLVFFAEDVGSNKGAIIGLMVGGVV-OH | Peptide Specialty Laboratories GmbH (Heidelberg) |
| Aβ(19-34) | H-FFAEDVGSNKGAIIGL-PEG-Biotin | Fraunhofer IZI (Halle, Germany) |
| pGlu3-Aβ(3-40) | H-(pE)FRHDSGYEVHHQKLVFFAEDVGSNKGAIIGLMVGGVV-OH | Fraunhofer IZI (Halle, Germany) |
| isoAsp7-Aβ(1-40) | H-DAEFRH(isoD)SGYEVHHQKLVFFAEDVGSNKGAIIGLMVGGVV-OH | BioCat GmbH (Heidelberg, Germany) |
| pSer8-Aβ(1-40) | H-DAEFRHD(pS)GYEVHHQKLVFFAEDVGSNKGAIIGLMVGGVV-OH | peptides & elephants GmbH (Hennigsdorf, Germany) |
| 3NTyr10-Aβ(1-40) | H-DAEFRHDSG(3NY)EVHHQKLVFFAEDVGSNKGAIIGLMVGGVV-OH | peptides & elephants GmbH (Hennigsdorf, Germany) |
| 3NTyr10-Aβ(1-16) | H-DAEFRHDSG(3NY)EVHHQK-NH2 | Fraunhofer IZI (Halle, Germany) |
| 3NTyr10-Aβ(6-14) | H-HDSG(3NY)EVHHC-NH2 | Fraunhofer IZI (Halle, Germany) |
| pGlu11-Aβ(11-40) | H-(pE)VHHQKLVFFAEDVGSNKGAIIGLMVGGVV-OH | Fraunhofer IZI (Halle, Germany) |
| pSer26-Aβ(1-40) | H-DAEFRHDSGYEVHHQKLVFFAEDVG(pS)NKGAIIGLMVGGVV-OH | peptides & elephants GmbH (Hennigsdorf, Germany) |
| isoAsp27-Aβ(1-40) | H-DAEFRHDSGYEVHHQKLVFFAEDVGS(isoD)KGAIIGLMVGGVV-OH | Fraunhofer IZI (Halle, Germany) |
| isoAsp27-Aβ(19-34) | H-FFAEDVGS(isoD)KGAIIGL-PEG-Biotin | Fraunhofer IZI (Halle, Germany) |
| isoAsp27-Aβ(23-33) | H-DVGS(isoD)KGAIIC-NH2 | Fraunhofer IZI (Halle, Germany) |

**Automated image analysis** **after single labeling immunohistochemistry**

In order to determine the stained plaque area after single labeling immunohistochemistry, representative ROIs of human brain sections were used for automated image analysis using Zeiss arivis cloud (**Suppl. Fig. 3**).


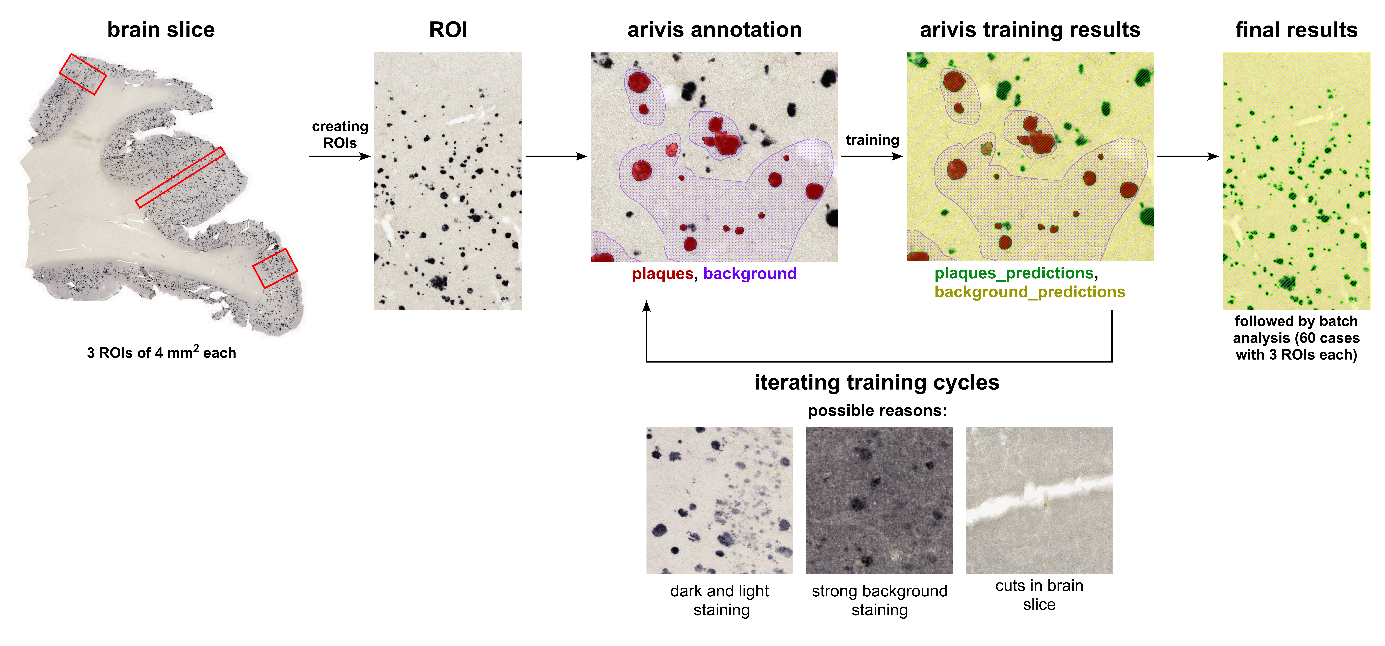


**Suppl. Fig. 3:** Automated image analysis with Zeiss arivis cloud after single labeling immunohistochemistry. For each case, three representative ROIs (red rectangles) of 4 mm^2^ each were placed in the gyri of the human brain slice and extracted using Zeiss ZEN 3.8 imaging tool. The width and length of the ROIs were adjusted to ensure that all ROIs were 4 mm^2^ and extended from the cortical surface to the white matter boundary. For each Aβ variant, a dataset of at least 12 ROIs of different cases and pathologies out of a total of 180 ROIs with a minimal image size of 1.024 x 1.024 pixels was uploaded to Zeiss arivis cloud. Two classes, plaques (shown in red) and background (shown in purple), were created and a selection of at least 50 objects per class were manually annotated. Care was taken to annotate different types and sizes of plaques as well as different background signals. After an initial training for semantic (pixel-based) segmentation, arivis training results (plaques_predictions in green and background_predictions in yellow) were evaluated by an experienced experimenter and refined by annotating more elements in both classes. The number of additional training cycles varied from one to four, depending on the intensity of plaque staining, background intensity or non-specific staining. The final trained model was used for batch analysis of all cases from all clinical conditions. For each Aβ variant, a separate training was performed with Zeiss arivis cloud due to different signal-to-background ratios and different staining patterns of the respective antibodies.

**Immunohistochemical detection of Aβ variants not present in amyloid plaques**

The antibodies against 3NTyr10-Aβ, pSer26-Aβ and isoAsp27-Aβ did not label amyloid plaques in control cases nor in any of the clinical conditions investigated (**Suppl. Fig. 4**). Instead, varying degrees of intracellular neuronal labeling were observed, with the greatest prevalence noted in layer V pyramidal neurons. For 3NTyr10-Aβ, almost no intracellular neuronal labeling in control cases and a weak immunoreactivity in the different clinical conditions was detected. The pSer26-Aβ antibody showed weak intraneuronal labeling in control, Pre-AD and VAD cases and a stronger labeling of individual pyramidal cells in AD and DLB cases (**Suppl. Fig. 4**). Immunohistochemistry using the isoAsp27-Aβ antibody revealed inconsistent staining of neuronal structures.

**Suppl. Fig. 4:** Representative images of immunohistochemical labelings for 3NTyr10-Aβ, pSer26-Aβ and isoAsp27-Aβ in control, Pre-AD, AD, DLB and VAD cases. Note the lack of association with amyloid plaques and the neuronal presence of these Aβ variants. The neuronal immunohistochemical labeling appears particularly strong for 3NTyr10-Aβ and pSer26-Aβ in individual pyramidal neurons.

**Quantification of Aβ variants not present in amyloid plaques by immunoassay**

The concentrations of the 3NTyr10-Aβ, pSer26-Aβ and isoAsp27-Aβ variants in the TBS and FA fractions were too low for quantification by immunoassay. Therefore, the respective protein concentrations were only displayed for the GdmCl fraction (**Suppl. Fig. 5a**). In general, all three modifications tended to be lower in AD cases than in the other clinical groups, with little fluctuation. We found no correlations between any of these Aβ variants and Braak stage, Thal phase and MMSE score (**Suppl. Fig. 5b**). Furthermore, the quantity of the three Aβ variants was low in all APOE genotypes (**Suppl. Fig. 5c**).


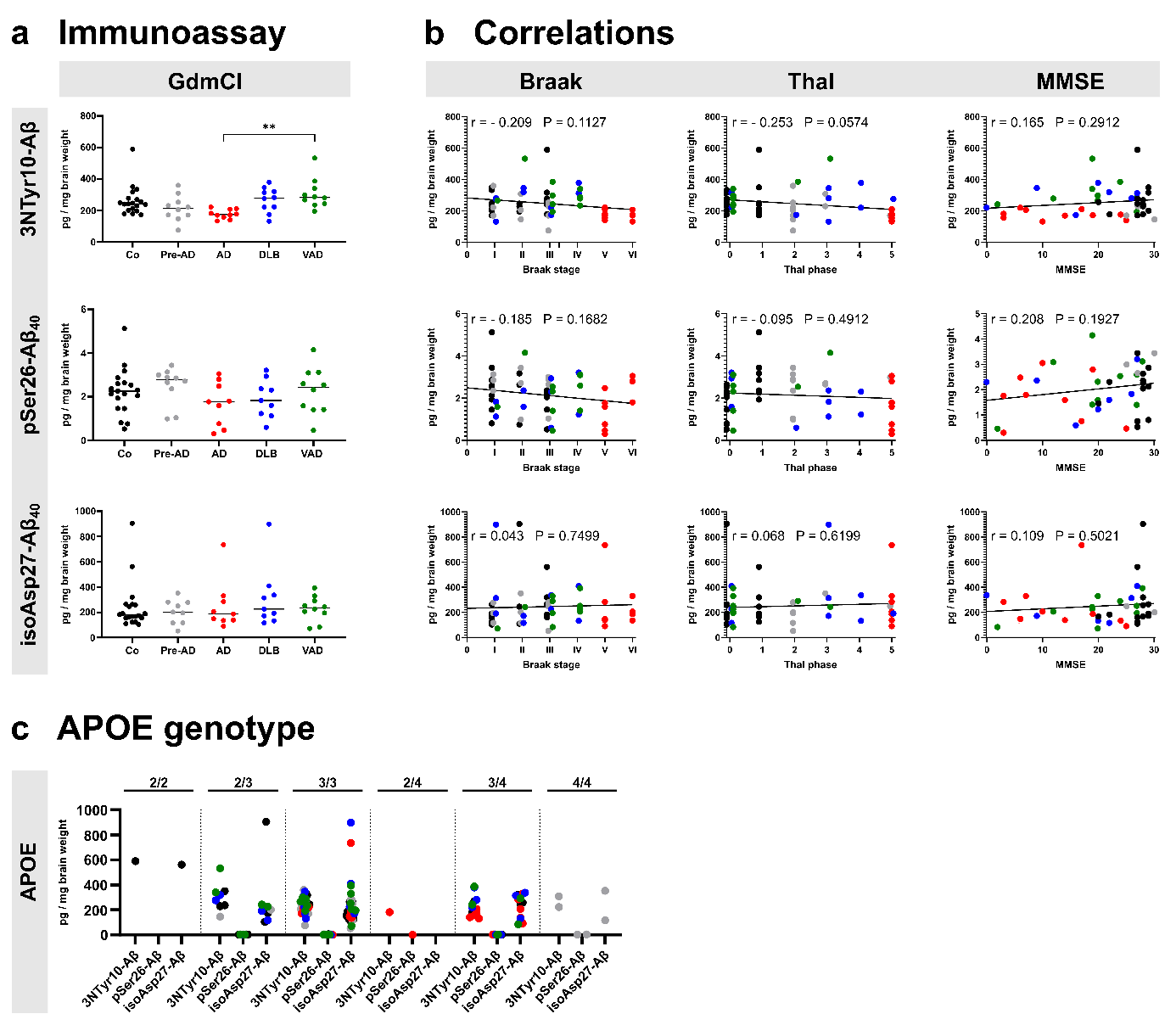


**Suppl. Fig. 5:** **(a)** Quantification of 3NTyr10-Aβ, pSer26-Aβ_40_ and isoAsp27-Aβ_40_ variants in control, Pre-AD, AD, DLB and VAD cases by immunoassay. In general, the amount of all Aβ variants in the AD cases tended to be lower than in the other clinical or control conditions. **(b)** Note the lack of correlation between Aβ concentrations in the GdmCl fraction and Braak stage, Thal phase and MMSE. **(c)** Subgroup analyses of the abundance of Aβ variants by APOE genotype. Note the low quantity of the three Aβ variants in all APOE genotypes. Missing individual values indicate measurements below the detection limit.

**Summary of immunohistochemical data and immunoassay quantifications**

Of the Aβ modifications investigated, immunohistochemical labeling revealed the highest plaque load for isoAsp7-Aβ in all clinical conditions, followed by pGlu3-Aβ in Pre-AD, DLB and VAD or Aβ(4-X) in AD (**Suppl. Fig. 6**). Immunoassay analyses showed comparable results, but isoAsp7-Aβ, pGlu3-Aβ and Aβ(4-X) exhibited greater similarity in their quantity (**Suppl. Fig. 6**).


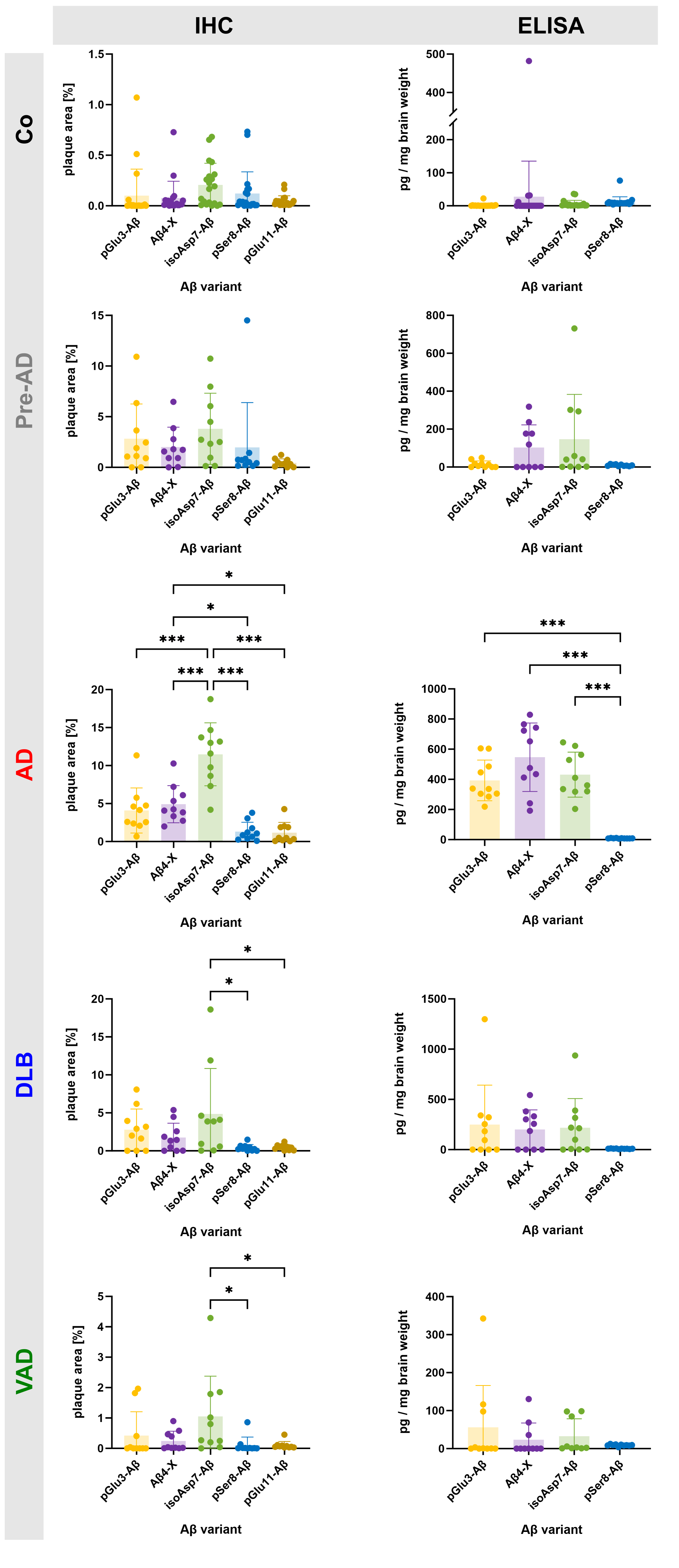


**Suppl. Fig. 6:** Summary data of the plaque load of Aβ PTMs detected with immunohistochemical labeling (left) and immunoassay quantification in the GdmCl fraction (right). Note the prominent plaque load and amount of isoAsp7-Aβ in all clinical conditions. Means and standard deviations are indicated by bars.

References

1. Sokolovsky M, Riordan JF, Vallee BL (1966) Tetranitromethane. A reagent for the nitration of tyrosyl residues in proteins. Biochemistry 5:3582–3589. https://doi.org/10.1021/bi00875a029
